# Green Hydrogen Production Technologies and Feedstock Limitations: A Concise Review

**DOI:** 10.1021/acsomega.6c06090

**Published:** 2026-09-15

**Authors:** Yasmeen Saleh, Ayesha Alam, Labeeb Ali, Abdulrahman Alraeesi, Mohammednoor Altarawneh

**Affiliations:** † 11239 United Arab Emirates University, Department of Chemical and Petroleum Engineering, Sheikh Khalifa bin Zayed Street, Al-Ain 15551, United Arab Emirates; ‡ United Arab Emirates University, College of Agriculture and Veterinary Medicine, Sheikh Khalifa bin Zayed Street, Al-Ain 15551, United Arab Emirates; § Abu Dhabi Polytechnic, Petroleum Engineering Technology Department, Abu Dhabi 111499, United Arab Emirates

## Abstract

Nowadays, intensive research is directed to green hydrogen production as an alternative to the depleting fossil fuel resources. Shifting from conventional hydrogen production routes, which rely on hydrocarbon reforming, toward cleaner and more sustainable pathways is a pivotal strategy to support decarbonization and minimize harmful emissions. Water and biomass are two abundant and sustainable resources that can serve as a promising green hydrogen feedstock. Water splitting into its constituents can be achieved by electrolysis, photocatalysis, and thermochemical techniques, while biomass can be converted into syngas through thermochemical processes, such as pyrolysis and gasification. Additionally, biomass has the potential to produce hydrogen through biological routes, like fermentation and microbial electrolysis processes. Electrolytic splitting of freshwater achieves a mass fraction of approximately 11.19% hydrogen (8.94 liters of water produces 1 kg of hydrogen). The highest energy efficiency of hydrogen (η_electrolyzer_) from the electrolyzer system has been reported in the literature to be 60–80% under a higher heating value with 3.35–5.6 kWh/Nm^3^ energy consumption. However, the utilization of desalinated water as an alternative feedstock is emerging as a strategic focus among global green hydrogen market participants. Integrating renewable energy resources, like solar and wind energy, into hydrogen production technologies is a critical approach that demands intensified research and development. This review discusses the major green hydrogen production technologies along with their fundamental characteristics, operating conditions, and the associated advantages and drawbacks. Furthermore, it provides an overview of recent concerns surrounding hydrogen production technologies and feedstock availability, while emphasizing the technical challenges that constrain the large-scale deployment of green hydrogen.

## Graphical abstract

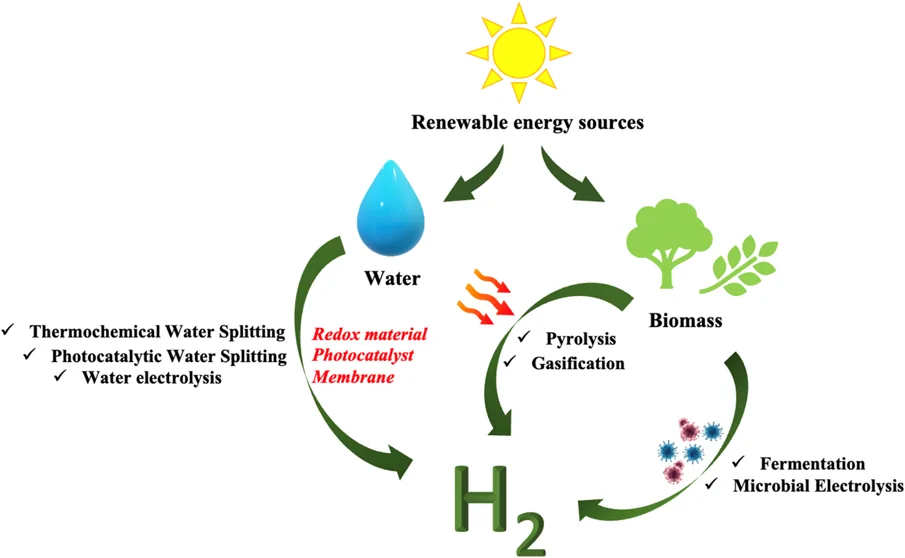

## 1 Introduction

The rising global energy demands are mainly driven by population growth and the industrial revolution, which have led to an increase in the atmospheric CO_2_ concentration from 280 to 421 ppm in 2022, causing severe environmental impacts, including climate change, global warming, loss of biodiversity, rising sea levels, and severe weather events such as floods and droughts.^1^ Significant efforts worldwide are currently underway to develop clean and sustainable energy sources to reduce GHG emissions and dependence on fossil fuels for energy generation.^2^ Shifting toward renewable energy sources such as solar and wind contributes to generating energy with minimal environmental impact. However, such resources are affected by location and weather variation, which lead to fluctuations in energy production.^3^ To overcome this constraint, energy generated from renewable resources can be harnessed by using storage technologies. Batteries are the most common storage devices that convert electrical energy to chemicals via reversible electrochemical reactions and enable decentralized energy storage. However, their use has technical limitations, including the reliance on polluting materials and difficulties in recycling.^4,5^ Therefore, hydrogen derived from renewable resources is gaining attention as a promising energy carrier that supports the shift toward a sustainable and decarbonized energy future. It offers diverse applications, including electricity generation through fuel cells, combustion for heat and mechanical power production, and a feedstock in industrial applications.^4,6^

In 1766, the British aristocrat Henry Cavendish found a low-density gas that surfaced to form water upon combustion in air. Afterwards, Antoine Lavoisier named this gas “hydrogen”, derived from the Greek terms’ hydro and genes’, based on its characteristic of forming water. Later, it was uncovered that hydrogen can be generated upon electrical splitting of water, a reverse chemical reaction of combustion.^7,8^ Different raw materials employed to different degrees generate a broad spectrum of hydrogen that intends to fulfill energy demand sustainably.^9^ As a result, hydrogen was employed broadly as a feedstock in chemical processing, predominantly in ammonia production by Haber’s process and purifying processes.^10^ A far its industrial part, hydrogen has appeared as a feasible energy haulier, with utilization in energy storage systems and transportation based on fuel cells.^11^ It is known that minimal environmental deterioration occurs when hydrogen is produced through electrolysis driven by renewable energy with low greenhouse gas emissions (GHGs), aligning it as a crucial constituent in the mitigation of climate change and carbon fluxes, as discussed earlier.^12^

The production of hydrogen through pathways that do not generate CO_2_ emissions is referred to as green hydrogen,^4,9^ which contributes to the achievement of key Sustainable Development Goals (SDGs), including SDG 7 (Affordable and Clean Energy) and SDG 13 (Climate Action).^6^ Methane steam reforming is the most common process for the production of hydrogen. However, it is associated with CO_2_ emissions and referred to as gray hydrogen.^13^ It is estimated that producing one ton of hydrogen through natural gas reforming results in the emission of approximately 14 tons of carbon dioxide; therefore, producing hydrogen from fossil fuels poses significant environmental challenges and does not offer a viable pathway toward sustainable energy development.^14^ Green hydrogen can be produced by a range of technologies, including water electrolysis, microbial electrolysis, photoelectrochemical and photocatalytic water splitting, solar thermochemical water splitting, direct water thermolysis, and arguably some biomass-based processes such as gasification and pyrolysis.^15^ Among these, electrolysis using electricity derived from renewable energy sources, such as wind and solar power, is the most promising for green hydrogen production, currently contributing to less than 4% of the total hydrogen production.^13^ Global green hydrogen production has increased from 37.1 kt in 2021 to 147.6 kt in 2023, with the Asia-Pacific countries accounting for the largest share at 93.6 kt.^16^ Several countries are actively advancing green hydrogen production. For example, Denmark is developing a “Hydrogen Island” powered by offshore wind in the North Sea, with the goal of achieving 300 GW of offshore wind capacity by 2050.^17^ Another large-scale hydrogen production project is currently under development in Germany, which aims to utilize wind energy for water electrolysis with a planned capacity of 10 GW.^18^ In China, a global market leader, multiple large-scale green hydrogen plants integrating solar and wind energy for electrolysis were developed.^19^ This review discusses green hydrogen production through water splitting and biomass thermochemical and biological conversion technologies, along with their characteristics, operating conditions, associated advantages and drawbacks, and limitations that hinder green hydrogen’s commercial-scale operation. It also discusses hydrogen storage and transportation along with technoeconomic analyses for various technologies.

## 2 Waste to Hydrogen

The rapid growth of the global population has increased the level of waste generation in the agricultural and food industries. These waste streams can be valorized as feedstock for hydrogen production through thermochemical conversion methods, such as pyrolysis and gasification, as well as biological routes, such as fermentation, serving as a sustainable energy source and an environmentally friendly waste management approach. Hydrogen derived from biomass can be considered CO_2_-neutral, as the CO_2_ released during the process was previously absorbed by the plants during their growth; therefore, it can be classified as green hydrogen.^4,20,21^ Integrating thermochemical biomass gasification with solar energy enables efficient conversion of biomass into hydrogen-rich syngas as solar heat supplies the reaction energy. This approach eliminates the need to burn part of the feedstock, conserving resources and preventing contamination from combustion byproducts, thereby enhancing green hydrogen generation.^22^ Abundant amounts of biomass are generated from agricultural fields, forests, food-processing industries, and livestock. For instance, beverage industries generate approximately 20–60% byproducts, while food processing industries convert about one-third of the fruits and vegetables produced annually into byproducts, according to estimates by the Food and Agriculture Organization (FAO) of the United Nations.^23,24^ In addition to biomass, plastic waste is accumulating, and it is expected that 12 billion tons will be dumped in landfills by 2050. This necessitates the implementation of effective waste management strategies to reduce environmental impacts and contribute to energy production. Biomass and plastic waste can serve as promising, sustainable sources for hydrogen production.^25,26^

While biomass has the potential for hydrogen production, its use is limited by feedstock properties and environmental impacts. Hydrogen yields depend on the biomass source, conversion process, and operating conditions.^27^ Economic costs associated with growing, harvesting, transporting biomass, and land use are additional concerns, as biomass-based hydrogen generally requires more land than fossil-based methods.^28,29^ Cultivating biomass can lead to deforestation and habitat loss if it is not managed carefully. Energy crops may compete with traditional agriculture, and algae cultivation demands significant land and water resources. Conversely, using agricultural and forest residues avoids additional land use, and converting municipal waste helps to reduce reliance on landfills. However, biomass gasification’s carbon footprint varies greatly due to different feedstocks and supply chain emissions, which can account for 25–87% of total emissions. Pretreatment can further increase the energy demand and associated emissions. Accordingly, feedstock availability, land use, and energy inputs are key factors affecting the reliability of biobased hydrogen pathways.^29,30^ Moreover, biomass degradation during storage results in feedstock and energy losses, increased economic costs, and greenhouse gas (GHG) emissions. Biological, chemical, and physical reactions within stored biomass promote microbial activity as pile temperatures rise, leading to the release of gases, such as CO_2_, CH_4_, and CO. Therefore, optimizing storage conditions throughout the biomass supply chain is essential to minimize these losses and improve the sustainability and economic viability of biomass utilization.^31^

### 2.1 Gasification

Gasification is a thermochemical technique that converts solid lignocellulosic biomass into syngas, which consists mainly of CO, CO_2_, H_2_, and CH_4_, in addition to biochar, tar, and ash. Subsequent separation and purification processes are required to recover hydrogen from syngas and produce high-purity hydrogen. Pressure swing adsorption (PSA), membrane separation, and syngas redox processes are among the most commonly used hydrogen recovery technologies.^32^ Gasification occurs within the temperature range from 500–1200 °C in the presence of gasifying agents (steam or air). Gasifying agents reform the biomass molecules, producing lower-molecular-weight gaseous products.^33,34^ The gasification process involves four main steps: drying (less than 150 °C), pyrolysis (150–700 °C), partial combustion (700–1500 °C), and reduction (800–1100 °C). In the drying step, moisture is removed from biomass. During pyrolysis, lignocellulosic materials (hemicellulose, cellulose, and lignin) are thermally degraded, producing gaseous products, tar, and char. The combustible products undergo oxidation, forming water vapor and CO_2_, which further react with the char, producing hydrogen and CO,^34,35^ as shown in Figure 1.^36^ Biomass gasification is one of the most attractive thermochemical technologies for green hydrogen generation due to the abundance and low cost of biomass feedstocks, which if utilized properly, can serve as a potential resource for meeting future green hydrogen demands.^37^

**Figure 1:**
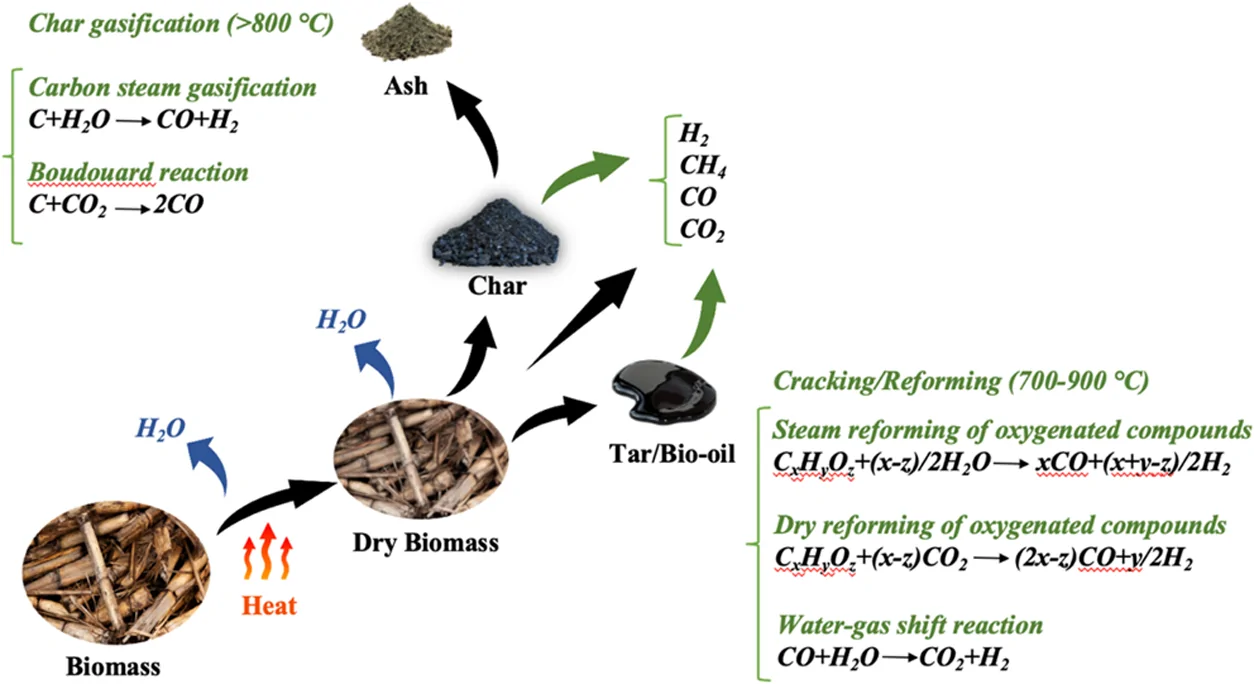
Schematic of biomass gasification for hydrogen production.

The hydrogen yield is influenced by the gasifying agent. For instance, using steam as the gasifying agent yields hydrogen in the syngas that is approximately three times higher than that obtained from air gasification.^25,38^ Saputro et al.^39^ developed an Aspen Plus model to assess the effects of catalyst type, steam to MSW ratio, and reactor temperature on syngas composition. The simulation showed that at a steam to MSW ratio of 0.5 at 900 °C, and using Fe_2_O_3_/Al_2_O_3_ and zeolite catalysts achieved the highest hydrogen yields (approximately 83.8%) with an optimal CO concentration of 14%. A similar study by Lan et al.^40^ was conducted on sawdust steam catalytic gasification, results showed that increasing temperature enhanced H_2_ and CO_2_ production while reducing CO and CH_4_ yields. Using steam as the gasifying agent raised hydrogen production by about 48%, and increasing the CaO catalyst to biomass ratio significantly enhanced H_2_ content in the produced syngas.

Gasification temperature positively affects hydrogen yield, with several studies identifying 700–800 °C as the optimal range for biomass catalytic gasification.^40^ Inayat et al.^41^ investigated H_2_-rich syngas production from steam catalytic gasification of empty fruit bunch biomass using CaO for CO_2_ capture. It was found that higher temperatures increased H_2_ and CO_2_ yields due to the endothermic nature of the reactions, while CaO presence promoted further H_2_ formation by capturing CO_2_ and driving the water-gas shift reaction forward. Biomass characteristics significantly influence syngas properties. For instance, high moisture content during biomass gasification enhances hydrogen production. Jahromi et al.^42^ investigated the gasification of sugarcane bagasse and found that increasing the moisture content led to higher H_2_ and CO_2_ production, attributed to the water-gas shift reaction and steam reforming. Variation in biomass composition, particularly in cellulose, hemicellulose, and lignin content, is mainly responsible for the differences observed in hydrogen yield.^43^ Tian et al.^44^ found that high lignin fraction in biomass tends to favor hydrogen production during gasification processes.

The heat required for conventional gasification is generated by partial feedstock combustion, which leads to higher pollutant emissions. Concentrated solar thermochemical gasification of biomass uses solar energy to drive the reaction, improving efficiency and reducing environmental impacts compared to conventional gasification.^45,46^ Solar energy offers significant potential for hydrogen production through concentrated solar power (CSP) systems, which provide the high temperatures needed for thermochemical reactions.^47,48^ To overcome the intermittent nature of solar energy, Bai et al.^49^ developed a novel solar-driven gasification approach employing solar-heated particles as heat carriers for biomass steam gasification in a fluidized bed reactor based on a computational particle fluid dynamics (CPFD) model. The results revealed that the syngas contained about 50% H_2_. However, large-scale applications remain limited by challenges such as particle heating, reactor design, and equipment costs. Arriagada et al.^50^ investigated the solar-driven gasification of char particles at low temperatures using a solar concentration system in a rotary hybrid porous media reactor. The numerical results showed that increasing the temperature enhanced the H_2_/CO production ratio, indicating the need for further studies of hydrogen and syngas production under higher-temperature operating conditions. Chuayboon et al.^51^ studied the high-temperature solar-driven gasification of beech wood biomass using a ZnO/Zn redox pair as a novel process producing both syngas and metallic zinc. As the temperature increased, H_2_ production rose significantly, CO production showed a slight increase, while CO_2_ and CH_4_ yields decreased.

### 2.2 Pyrolysis

Biomass pyrolysis is the thermal degradation of lignocellulosic biomass in an oxygen-deficient environment, producing biochar, bio-oil, and pyrolytic gases. It is classified based on the applied heating rate and residence time. Slow pyrolysis (carbonization) occurs at temperatures below 500 °C with long residence times and primarily targets char production. Fast pyrolysis operates at higher heating rates (10–200 °C/min) and shorter residence times (<2 s), yielding a high fraction of bio-oil. Flash pyrolysis takes place at extremely high heating rates (10^3^–10^4^ °C/s) with very short residence times (<0.5 s), resulting in bio-oil yields of about 75–80%.^52,53^

Quantifying hydrogen production from biomass pyrolysis is essential for evaluating the efficiency of this process as a hydrogen generation pathway. Several key factors influence hydrogen yield, and understanding these parameters is crucial for optimizing the process.^54^ Hydrogen yield increases with rising pyrolysis temperature, and the use of catalysts such as Al_2_O_3_, CaO, Mo_2_C, FeO, MnO, Cr_2_O_3_, CuO, and Na_2_CO_3_ further enhances H_2_ production by suppressing tar formation.^55^ Hu et al.^56^ investigated bamboo pyrolysis for H_2_ production through pyrolysis over the bifunctional phase change catalyst NiO-Ca_9_Co_12_O_28_. Results showed that raising the pyrolysis temperature from 550 to 650 °C led to a significant increase in hydrogen yield, from 213.09 to 361.68 mL/g, due to enhanced secondary tar cracking, while temperatures above 650 °C reduced H_2_ production because high CO levels favor the reverse methane reforming reaction. Another study by Yan et al.^57^ investigated the pyrolysis of tobacco stems in an induction-heating fluidized bed reactor to produce hydrogen-rich syngas. The results indicated that the yields of H_2_ and CO increased rapidly up to 550 °C, followed by a gradual rise to 700 °C. It was suggested that the polycondensation of char at higher temperatures produced CO and H_2_.

Ni-based steam reforming catalysts, which are commonly used in the petrochemical industry for methane and naphtha reforming, have shown excellent performance in biomass gasification, bio-oil steam reforming, and in-line reforming of pyrolysis volatiles.^58^ Wang et al.^59^ utilized biochar derived from poplar pyrolysis as a catalyst. Both raw char and Ni-loaded char catalysts with varying nickel contents were tested for the production of hydrogen-rich syngas from poplar wood. The Ni/char catalyst exhibited superior catalytic activity compared with the raw biochar. The total syngas yield increased with nickel loading up to 10 wt %, beyond which it declined due to pore blockage and reduced active sites caused by excessive Ni loading. A recent study by Zhang et al.^60^ examined bagasse catalytic pyrolysis, revealing that metal salts strongly affect product distribution and H_2_ yield. Ni salts showed the highest catalytic activity, maximizing H_2_ production and optimizing the gas composition, while Na and K reduced bio-oil formation. The optimal Ni loading is 5 wt%, beyond which gas yields decrease due to catalyst saturation. Microwave heating offers several advantages over conventional methods, including rapid, selective, and volumetric heating, noncontact energy transfer, and quick start-up and shutdown. It efficiently heats materials internally without wasting energy on bulk heating, which enhances safety.^61^ Zhang et al.^62^ conducted microwave-assisted pyrolysis of bagasse in a custom quartz reactor using Ni/SiC as a catalyst. The hydrogen yield increased with increasing Ni content up to 8%, beyond which both hydrogen production and bio-oil conversion efficiency declined. Similarly, increasing the microwave power enhanced the H_2_ content, reaching a peak of 51.04% at 1000 W, with the growth rate slowing beyond 800 W.

Solar energy can be integrated as the heating source to drive biomass pyrolysis for green fuel production.^63^ Arribas et al.^64^ studied the solar-driven pyrolysis and gasification of three different biomass types using a high-flux solar simulator. The results revealed that gasification outperformed pyrolysis, producing higher concentrations of syngas (CO and H_2_). Table 1 summarizes recent studies on thermochemical processes for hydrogen production.

**Table 1:** Recent Studies on Biomass Thermochemical Conversion for Hydrogen Production

thermochemical process	biomass type	study objective	main findings	references
gasification	pine chips	to study the effect of heat released during CO_2_ capturing by CaO on hydrogen yield during steam gasification	H_2_ yield increased and tar yield decreased compared to without CaO	^65^
gasification	pine sawdust	to investigate the effect of different Ni loadings catalyst and CaO adsorbent on biomass steam gasification	the highest hydrogen and the lowest CO_2_ yields were obtained at 2:1 Ni/Ce ratio at 600 °C	^66^
gasification/hybrid system	NA	proposing a hybrid system combining solar pyrolysis followed by gasification by steam or oxygen produced from photovoltaic water electrolysis, while the produced hydrogen used to adjust syngas composition	an increase of 10.45% in energy efficiency in comparison to conventional gasification	^67^
solar gasification	rice husk	to propose a novel-solar-driven gasification system that produces syngas and pure hydrogen in separate streams	the proposed gasification system is a promising approach for high-purity hydrogen and syngas production in separate streams	^68^
solar gasification	MSW	to propose a novel gasification system combining concentrated solar power and thermal energy storage, utilizing Ca looping for CO_2_ capturing, to produce pure hydrogen and electricity.	- the system produced 0.58 kg/s pure H_2_	^69^
			- Co_2_ capture efficiency is 95%	
			- 13.96 MW net electricity was generated	
solar gasification	wheat straw	to develop a novel direct irradiation solar gasification system consisting of a high-flux solar simulator and a gasification reactor	- increasing steam flow increased H_2_/CO ratio	^70^
			- smaller biomass particles improved the conversion	
solar pyrolysis	waste wood, waste straw, and sewage sludge	to investigate the solar pyrolysis of three waste types in a fixed bed reactor exposed to concentrated radiation by a Xenon lamp	H_2_ production was dominant at the final pyrolysis temperatures for the three samples	^71^
pyrolysis	bamboo	to investigate hydrogen production via pyrolysis of bamboo and carbide slag employing perovskite catalysts, and investigating different Ni doping levels on LaCoO_3_ catalyst	the highest H_2_ yield was obtained at 600 °C and at a mass ratio of bamboo to carbide slag of 5 to 15, using Ni_3_La_2_Co_2_	^72^

### 2.3 Fermentation

#### 2.3.1 Dark Fermentation

Dark fermentation is a biological process for converting organic waste into biohydrogen that is carried out under anaerobic conditions in the absence of light. In this process, heterotrophic bacteria act as biocatalysts, facilitating the degradation of the organic matter. Essentially, dark fermentation can be considered a truncated version of anaerobic digestion (AD).^73,74^ Clostridia are the primary hydrogen-producing bacteria. As Gram-positive, spore-forming microorganisms, they play a crucial role in biohydrogen production, naturally generating hydrogen at high rates through mixed-acid fermentation.^75^ The dark fermentation process starts with glycolysis, where glucose or other sugars are broken down to pyruvate. The resulting end products vary depending on several factors, such as the microbial species involved, the type of substrate, pH, temperature, oxygen sensitivity, and hydrogen partial pressure.^73,76^ As shown in Figure 2, facultative anaerobes, such as *Enterobacteriaceae*, further convert pyruvate into acetyl-CoA and formate via pyruvate formate lyase (PFL), with formate subsequently decomposing into hydrogen and carbon dioxide by formate hydrogen lyase (FHL). In obligate anaerobes such as *Clostridium* species, pyruvate is oxidized to acetyl-CoA and carbon dioxide through the action of pyruvate ferredoxin oxidoreductase (PFOR), which simultaneously reduces ferredoxin. The reduced ferredoxin (Fd_red_) is reoxidized by [Fe–Fe] hydrogenase (HydA), which catalyzes the release of molecular hydrogen during the process.^74,75,77^

**Figure 2:**
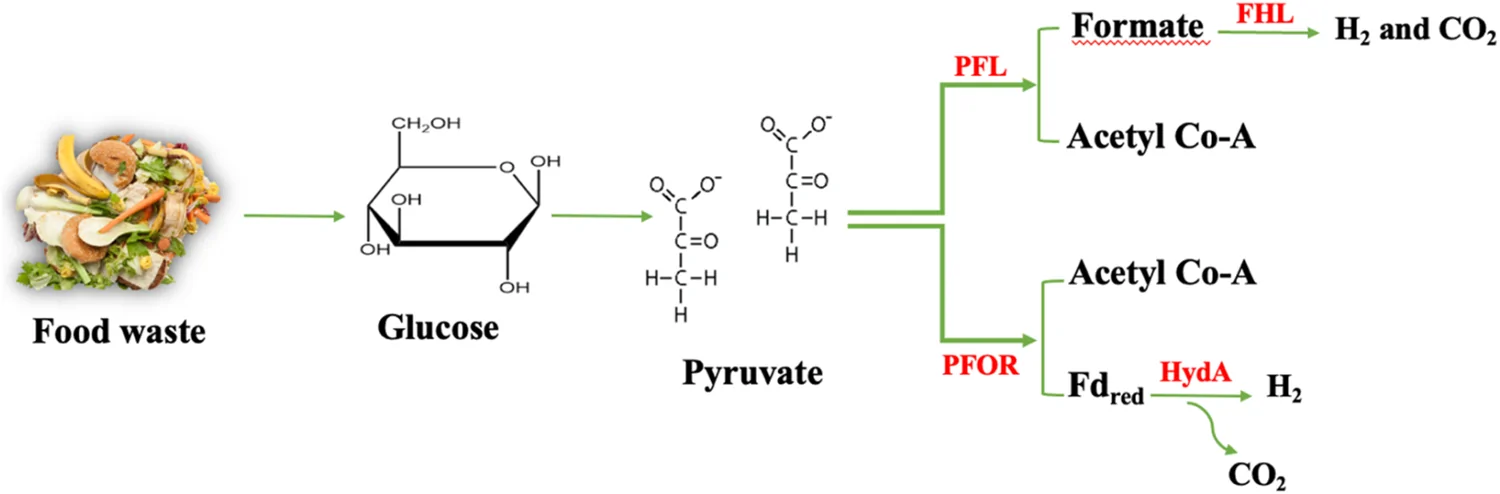
Dark fermentation for hydrogen production. Adapted with permission from ref 77 Copyright 2018, Elsevier.

The fermentation process is affected by many factors. The temperature should remain within the optimal range for the specific microbial species as conditions outside this range can hinder hydrogen production. A slightly alkaline environment is favorable for *Clostridium* species, enhancing their biohydrogen-generating activity. Increasing the substrate concentration can also promote bacterial growth by providing more nutrients and energy, thereby improving hydrogen yields. Among various substrates, glucose demonstrates the highest biohydrogen-production efficiency. However, fermentation of complex substrates such as starch, lignocellulosic materials, lipids, and protein-rich wastes typically results in lower yields due to the complex structural composition of the substrates, the rigid structure of lignin, the poor solubility and slow hydrolysis of protein-based materials, and the presence of long-chain fatty acids and phenolic compounds that suppress microbial activity.^77,78^ To accelerate fermentation kinetics, a hydrolysis pre-treatment is typically employed to depolymerize complex substrates into fermentable sugars such as glucose and maltose before hydrogen production. However, this additional step increases process complexity, as well as capital and operational costs.^77,78^ The major drawback of dark fermentation using food waste is its low hydrogen yield, which limits its industrial feasibility.^79^ Yang et al.^80^ showed that adding Fe-modified biochar (Fe-L600) notably enhanced hydrogen production during thermophilic dark fermentation of food waste, resulting in a 31.19% increase in hydrogen yield. Tang et al.^81^ investigated the effect of pH on hydrogen production from high-concentration nitrogen-containing organic wastewater and found that an initial pH of 5 provided optimal conditions for both hydrogen generation and denitrification. At pH 4-7, *Clostridium sensu stricto 12 sp*. dominated as the main hydrogen producer, while at pH 8-11, *Clostridium sensu stricto 1 sp*. was the predominant hydrogen-producing bacterium. Wongfaed et al.^82^ studied the enhancement of dark fermentative hydrogen production using Fe- and Ni-modified biochar derived from sugarcane bagasse and sugarcane leaves. Metal modification improved the surface properties of biochar and selectively enriched hydrogen-producing bacteria.

#### 2.3.2 Photofermentation

Photofermentation is a process in which purple non-sulfur bacteria (PNSB) utilize solar energy to convert organic feedstocks, such as simple carbohydrates, low-molecular-weight fatty acids, and industrial or agricultural residues, into hydrogen in the absence of oxygen, providing a sustainable and clean energy production method.^83,84^ PNSB exhibit remarkable metabolic flexibility, capable of growing both as photoautotrophs and chemoheterotrophs. Unlike plants and algae, they do not depend on water for their photosynthesis. They use inorganic ions, carbon compounds, or hydrogen as electron sources with energy generation driven by photosynthesis. PNSB can metabolize a wide variety of organic carbon compounds, including pyruvate, acetate, amino acids, alcohols, organic acids, and carbohydrates.^83^ Photofermentation for hydrogen production uses photosynthetic bacteria (mainly *Rhodobacter sphaeroides*, *Rhodopseudomonas palustris*, and *Rhodobacter capsulatus*) to convert organic substrates to hydrogen in the presence of light. As depicted in Figure 3, organic feedstocks are converted via the tricarboxylic acid (TCA) cycle into protons, electron donors, and CO_2_. Light energy excites electrons, producing high-energy electrons that follow two pathways: some transfer to ferredoxin and then to nitrogenase for hydrogen production, while others move through the electron transport chain to generate adenosine triphosphate (ATP).^85^

**Figure 3:**
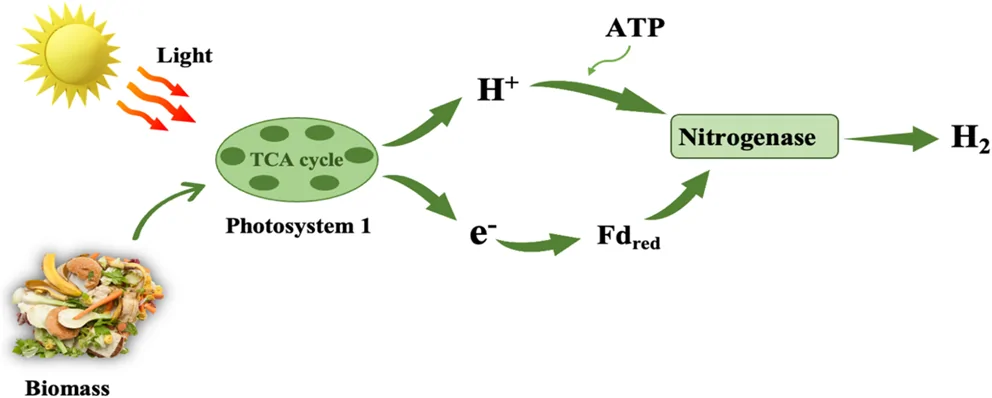
Biomass photofermentation process for hydrogen production. Adapted with permission from ref 85 Copyright 2021, Elsevier.

Photofermentation is affected by several key factors, such as the substrate type (e.g., glucose, sucrose, acetate), the support material properties (such as porosity and texture), as well as environmental conditions like pH, temperature, light source, and intensity, all of which affect bacterial activity and hydrogen production efficiency.^86^ Shui et al.^87^ investigated the effect of light fluctuation on hydrogen production from giant reeds photofermentation. It was found that elevating the light intensity from 3000 to 6000 Lux for 24 hours resulted in 123 mL/g TS hydrogen yield, which was higher than the yields achieved under light fluctuation at 36 hours. Ren et al.^88^ developed a 70 L composite tubular photobioreactor to study photofermentative hydrogen production using glucose in both batch and continuous modes under outdoor conditions. Coupling artificial light with sunlight enhanced hydrogen production efficiency, and an optimal hydraulic retention time of 5 days was achieved in continuous operation. The system demonstrates the potential of continuous photofermentation for large-scale photobiological hydrogen production. Yue et al.^89^ evaluated the hydrogen production potential of eight types of landscaping wastes. *Buxus megistophylla* was identified as the best substrate for hydrogen production via photofermentation. Under optimal conditions 29.78 °C, 3000 Lux, 21.49 g/L substrate concentration, and initial pH 6.78, the highest cumulative hydrogen yield was 73.82 mL/g TS. Zhang et al.^90^ proposed adding nano-TiO_2_ to corn straw photofermentation, which increased hydrogen yield by 32.6%. Nano-TiO_2_ addition enhanced the degradation of butyric acid and acetic acid, thereby improving hydrogen production from corn straw photofermentation.

### 2.4 Microbial Electrolysis Cell (MEC)

Microbial electrolysis cells (MECs) are a biological hydrogen production technology offering a novel approach to generating clean and sustainable hydrogen from carbon-rich feedstocks such as sewage water, biomass, and waste from treatment facilities.^91,92^ As shown in Figure 4, the system comprises an anode, electrogenic microbes, a membrane, a cathode, and an external power supply. In MECs, organic compounds are oxidized in the anode chamber, while hydrogen is produced in the cathode chamber through electrohydrogenation. During this process, exoelectrogenic microbes release electrons that combine with protons to form hydrogen gas, unlike microbial fuel cells (MFCs), which generate electricity instead.^93^ The hydrogen yield is directly proportional to the current produced in the anaerobic chamber.^94^ MEC technology offers several advantages, including a high hydrogen yield, wide substrate versatility, low energy consumption, and simultaneous waste-to-energy conversion. However, its large-scale application remains constrained by the high cost of metal catalysts, low hydrogen purity, and hydrogen losses due to methanogenic activity in mixed-culture systems.^91^ A recent study by Guerrero-Sodric et al.^95^ successfully demonstrated a 150 L MEC pilot plant for sugar-based industrial wastewater treatment, employing nickel-foam and stainless-steel wool cathodes as catalysts. The system achieved a hydrogen production rate of 19.07 L m^–2^ day^–1^, a high current density of 2 A m^–2^, and an organic matter removal efficiency of 40%. Chavan et al.^96^ utilized bamboo biomass for clean hydrogen production through successive enzymatic treatment followed by microbial electrolysis in an MEC. The system employed an anode modified with monodisperse iron oxide nanoparticles (IONPs), which enhanced the conversion efficiency, achieving a hydrogen production rate 1.14 times higher than that of the uncoated anode. Rossi et al.^97^ demonstrated that a low-cost hydrothermally synthesized NiMo cathode catalyst can effectively replace noble metals for hydrogen production in MECs. Using this catalyst, hydrogen was generated at a high rate of approximately 81 L H_2_ L^–1^ day^–1^. The strong performance and low cost of the NiMo catalyst highlight its potential as an economically viable option for advancing biohydrogen generation in MEC systems. Table 2 lists recent studies on biological processes for hydrogen production.

**Table 2:** Recent Studies on Biomass Biological Processes for Hydrogen Production

biological process	biomass type	study objective	main findings	references
dark fermentation	cassava pulp and cassava processing wastewater	to investigate hydrogen production via dark fermentation in a 2L bioreactor	the obtained H_2_ yield was 83.1%, offering high solid dark fermentation as a promising process for H_2_ production	^98^
MEC and dark fermentation	swine manure and food waste	to propose MEC and dark fermentation integrated system for hydrogen production	- the proposed system mitigated the high voltage and low H_2_ yield challenges of the conventional MEC process	^99^
			- the proposed system achieved four times higher H_2_ production	
dark fermentation	paper mill effluent	to explore H_2_ production from untreated paper mill discharge in a packed bed reactor at different hydraulic retention time (HRT) and organic loading rates	the highest H_2_ production rate was obtained at HRT of 2 h, and the maximum yield was attained at HRT of 8 h	^100^
photofermentation	corn stover	to investigate corn stover photofermentation for hydrogen production using CaTiO_3_ photocatalysts doped with Fe	adding 6% Fe-doped CaTiO_3_ improved the cumulative H_2_ production by 63.4%, H_2_ production rate by 203.4%, conversion, and shortened H_2_ production lag phase from 17.1 to 11.1 hrs	^101^
photofermentation	corn cob	to optimize the operating conditions for hydrogen production by photofermentation using response surface method (RSM) and a genetic algorithm-optimized neural network (GANN)	- GANN model performed better in prediction. however, its long-term stability requires more investigation	^102^
			- RSM demonstrated the interactions between the studied factors	
			- factors like temperature and light intensity were not investigated and require further research	
photofermentation	corn stover	to introduce advanced convolutional neural networks (CNN) and long short-term memory networks (LSTM)-Attention model to optimize hydrogen production via photofermentation, addressing challenges like process non-linearity	- the proposed model outperformed the traditional machine learning models in accuracy	^103^
			- at the optimum conditions, H_2_ production rate was 42.31 mL/h	
photofermentation	glucose	to investigate the batch and continuous hydrogen production via photofermentation using a novel 70 L tubular photobioreactor in an outdoor environment	the highest H_2_ production rate was 22.8 mL H_2_/(L·h), at 5 days hydraulic retention time during the continuous process, and 37.6 mL H_2_/(L·h) in the batch process.	^88^
MEC	wastewater	introducing Na in MEC system for salt-containing wastewater treatment and hydrogen production	Na presence contributed to improving electron transfer, which enhanced the organic pollutant decomposition at the anode to 91.34%, while improving H_2_ production by 1.57 to 1.70 times	^104^
MEC	poplar leaves and wastewater sludge	to explore membraneless MEC for hydrogen production using nanomaterials (Al_2_O_3_, MgO, and Fe_2_O_3_ metal oxides) and wastewater sludge as an inoculum resource.	- the highest H_2_ production of 828 mL was obtained by adding 1 gr of Fe_2_O_3_ metal oxide	^105^
			- the decrease in inoculum amounts reduced H_2_ production	
MEC	wastewater	to propose a 1 m^3^ MEC pilot plant fed with wastewater for hydrogen production.	- the highest H_2_ production rate was 8.59 L m^–2^ d^–1^ for synthetic wastewater and 3.45 L m^–2^ d^–1^ for real wastewater	^106^
			- the proposed system showed good scalability. however, reducing material cost, H_2_ leakage, and voltage loss requires further investigation	

**Figure 4:**
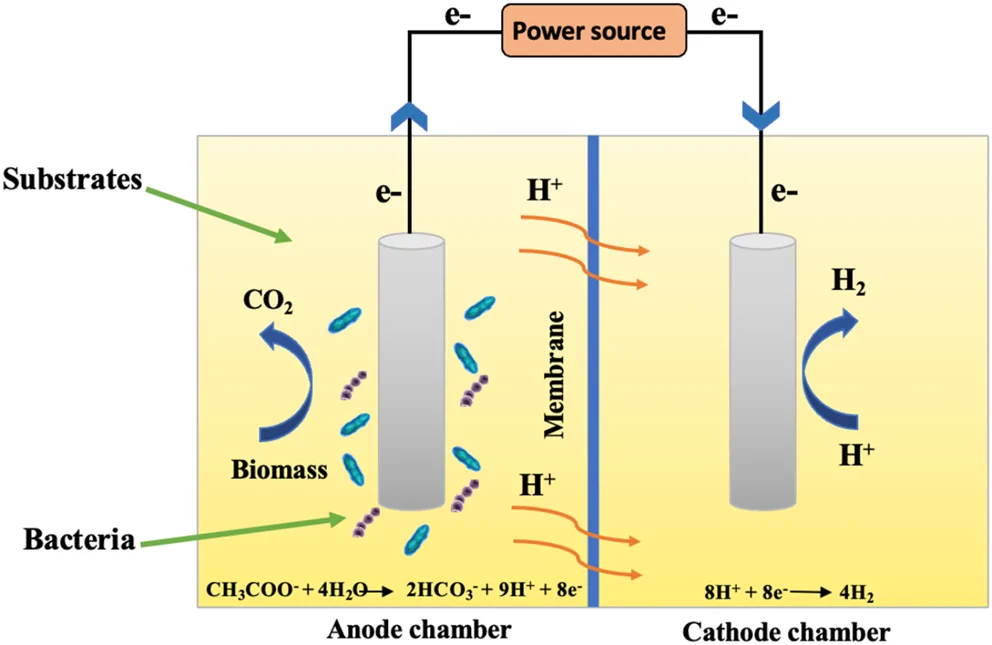
Schematic of a microbial electrolysis cell (MEC). Adapted with permission from ref 92 Copyright 2016, Elsevier.

## 3 Water to Hydrogen

Green hydrogen can be produced from water splitting by using heat, electricity, or light derived from renewable energy sources. For example, wind energy can be converted to electricity to drive water electrolysis. Solar radiation can split water in the presence of photocatalysts through photo- or photocatalytic processes. In addition, solar energy can be harnessed through concentrated solar power (CSP) technologies to achieve the elevated temperatures required for thermal water splitting.^107^

### 3.1 Water Electrolysis

Water electrolysis is a process to produce hydrogen by splitting water into hydrogen and oxygen by using an electric current. It is a high-potential technology for generating green hydrogen using electricity derived from renewable energy sources, including solar, wind, hydropower, and geothermal energy.^108^ Ions at the electrodes gain or lose electrons, and the process can yield hydrogen with up to 99.999% purity after removing oxygen. However, the technique is costly compared with fossil-based methods. Electrolysis occurs in a cell with two metallic electrodes separated by an ionic conductor, where an applied direct current drives electron flow by altering the electrode potentials. Water is supplied to the cell, and hydrogen and oxygen are collected separately at the outlets.^109,110^ Water electrolysis can be implemented using various types of electrolyzers, mainly including alkaline water electrolysis (AWE), proton-exchange membrane (PEM), solid oxide electrolysis (SOE), and anion-exchange membrane (AEM). Among these technologies, AWE cells are the most mature but have a relatively low efficiency, prompting the development of alternative methods. PEM and AEM electrolyzers offer higher efficiency and a more compact design than alkaline systems, but they rely on costly materials, leading to higher capital costs. SOE cells are the most efficient, yet they operate at high temperatures and are the most expensive option.^15^

#### 3.1.1 Alkaline Water Electrolysis (AWE)

Electrochemical water splitting involves two half-reactions: the hydrogen evolution reaction at the cathode and the oxygen evolution reaction at the anode, typically separated by an ion-exchange membrane. These reactions are controlled by factors such as temperature, pressure, electrolyte type and pH, electrode material, and cell configuration. As depicted in Figure 5a, under alkaline conditions, hydroxide ions generated at the cathode serve as charge carriers; their oxidation at the anode produces oxygen and electrons, which are then used to generate hydrogen at the cathode. The reactions occur at the electrode-electrolyte interface, and the gas evolution rate is proportional to the electric current according to Faraday’s law of electrolysis.^111^ An electrochemical cell typically requires an external power supply to initiate water splitting. To improve conductivity, acidic or alkaline electrolytes are added, as pure water has low electrical conductivity at room temperature. In alkaline water electrolysis, potassium hydroxide (KOH) or sodium hydroxide (NaOH) is commonly used. KOH dissociates into ions that conduct electricity between the electrodes. The electrodes, usually made of nickel, are separated by an ion-conductive membrane that allows hydroxide ion (OH^‑^) transport while preventing gas crossover.^112^

**Figure 5:**
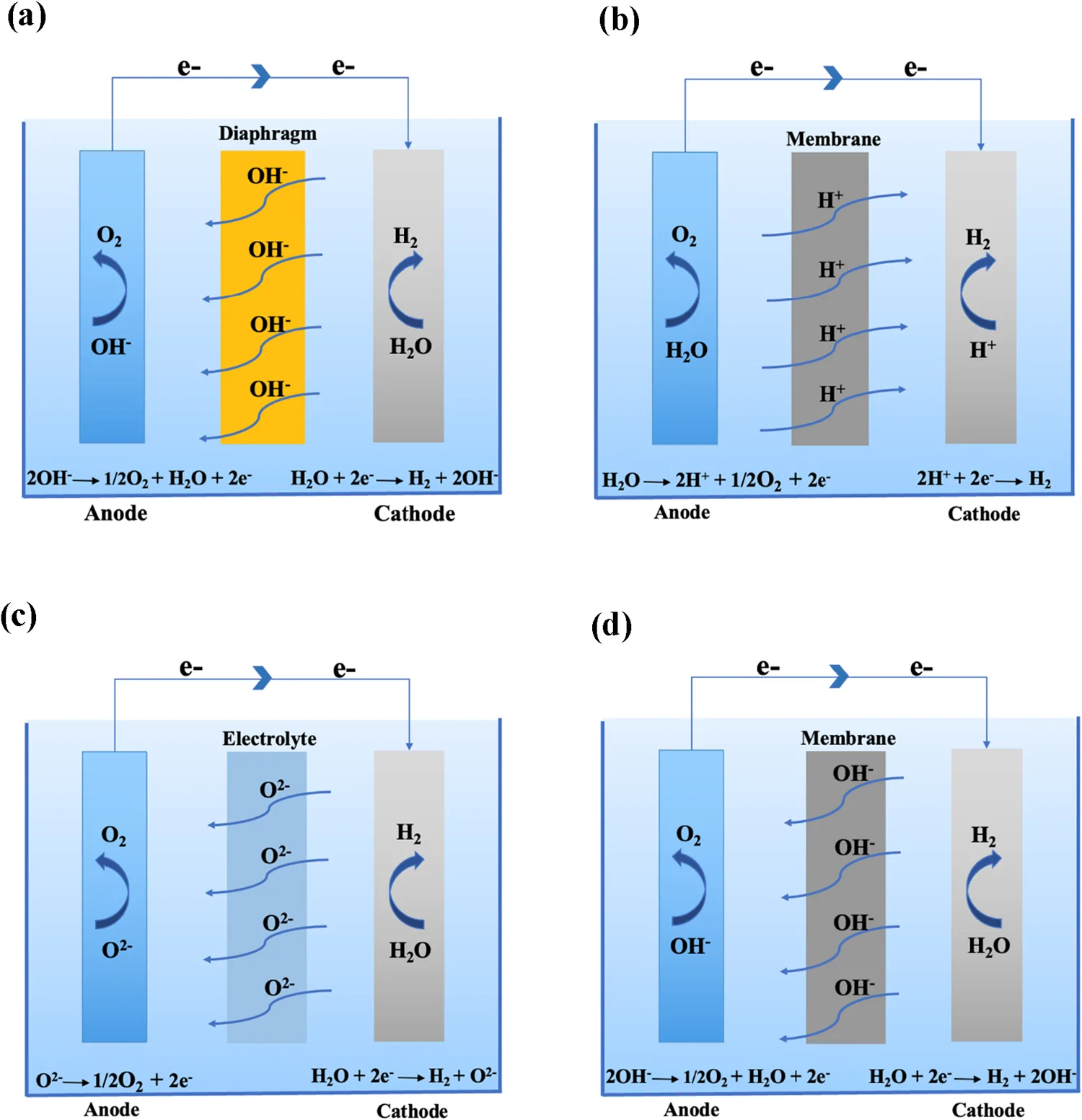
Schematics of (a) alkaline water electrolysis (AWE), (b) proton exchange membrane (PEM), (c) solid oxide electrolysis (SOE), and (d) anion exchange membrane (AEM).

A recent study by Bora et al.^113^ investigated a 25-kW alkaline electrolyzer coupled with a hybrid PV-grid power system. Despite the intermittent voltage, the electrolyzer demonstrated stable operation with consistent hydrogen output. Results revealed that the stack voltage remained steady at 37.8 °C and 52 A, achieving a maximum efficiency of 65%. Another study by Marceta Kaninski et al.^114^ introduced the integration of a permanent magnetic field within situ activation employing cobalt-based complexes and molybdenum salt, which improved electrolyzer performance. This approach reduced energy consumption by approximately 38% and enhanced overall efficiency by eliminating the conventional electrode deposition step. Alkaline water electrolysis is an established and cost-effective technology for hydrogen production, with several commercial systems currently available.^115^ It utilizes inexpensive nickel-based catalysts and offers relatively low capital costs compared to other electrolysis systems. However, it also presents some limitations, such as the use of highly corrosive electrolytes that pose safety risks and contribute to material degradation, low current density, and lower overall energy efficiency.^108,116–118^

#### 3.1.2 Proton Exchange Membrane (PEM)

In a PEM electrolyzer, water is supplied to the anode, where it is split into protons (H^+^ ions) and oxygen. The protons then migrate through the proton-conducting membrane to the cathode, where they combine to form hydrogen gas, while the oxygen remains at the anode side with the residual water, and electrons flow from the anode through the external power circuit, supplying the driving force (cell voltage) necessary for the reaction,^110,119^ as shown in Figure 5b. PEM water electrolysis technology employs solid polysulfonated membranes as electrolytes that conduct protons which offer several advantages, such as low gas permeability, high proton conductivity (0.1 ± 0.02 S cm^–1^), reduced thickness (approximately 20–300 μm), and suitability for high-pressure operation. The electrocatalysts used in PEM electrolysis are noble metals like Pt or Pd for the hydrogen evolution reaction at the cathode and IrO_2_ or RuO_2_ for the oxygen evolution reaction at the anode, which makes the technology more expensive than alkaline water electrolysis.^119^

Proton exchange membrane electrolysis offers notable advantages, including operation at high current densities, flexible design, and rapid response to varying power inputs, making it highly suitable for integration with variable renewable energy sources.^108,117^ However, it also faces challenges such as the high cost of metal catalysts like platinum and iridium, and a shorter lifespan compared to alkaline systems.^117^ Recently, extensive research has focused on optimizing various parameters of PEM electrolyzers, including material composition, stack design, operating conditions (e.g., current density and temperature), water and gas management, durability, and maintenance. Novel materials have also been developed for the catalyst layers, proton exchange membranes, and gas diffusion layers to improve overall system performance.^120,121^ Li et al.^121^ optimized PEM membrane thickness using the sequential quadratic programming (SQP) algorithm to produce hydrogen efficiently and safely, with the objective of increasing the hydrogen production rate while ensuring that the hydrogen concentration on the anode side did not exceed 2%. It was found that at an input power of 1800 W, the optimal membrane thickness was 28 μm. The results indicate that determining the membrane thickness depends on both the average solar radiation intensity and its fluctuations.

Wilberforce et al.^122^ generated green hydrogen using a polymer electrolyte membrane electrolyzer powered by a vertical-axis wind turbine. It was found that the electrolyzer performed best at high temperatures and low pressures, with wind intensity directly related to the electrolyzer performance. Under the tested conditions, at 1 bar pressure and an operating temperature of 20 °C, the system produced 65,770 L of hydrogen. Huang et al.^123^ proposed a novel Ag@SiO_2_ nanofluid-filtered photovoltaic/thermal system for H_2_ and freshwater production from seawater. They investigated the effects of mass flow rates and nanofluid parameters on system performance. The results revealed that the system is more sensitive to nanofluid flow rate than to coolant flow rate, achieving H_2_ production rate of 1.95 × 10^–5^ kg/s and overall system efficiency of 22.39%. AI can be integrated into water electrolysis technologies to enhance the process performance. Tawalbeh et al.^124^ explored the integration of advanced machine learning (ML) techniques to improve the efficiency and operational performance of hydrogen production through PEM. The study demonstrated that artificial neural network (ANN) models possess superior predictive capabilities compared to traditional regression algorithms, making them highly effective for accurately forecasting hydrogen production rates.

#### 3.1.3 Solid Oxide Electrolysis (SOE)

Solid oxide electrolysis operates at high temperatures and pressures, typically above 600 °C, with water supplied to the cell in the form of steam. As depicted in Figure 5c, the steam is introduced to the porous cathode, where it dissociates into hydrogen and oxygen ions at the cathode-electrolyte interface. The generated hydrogen gas diffuses outward and is collected at the cathode surface, while the oxygen ions transfer through the dense electrolyte to the anode, where oxidation occurs to form oxygen gas, which then diffuses through the porous anode to the surface for collection.^125,126^ High-temperature solid oxide electrolysis outperforms other types of water electrolyzers, as it overcomes many challenges associated with seawater electrolysis. Seawater contains salts and impurities that can corrode the electrolyzer, and another major issue is the competition at the anode between the chlorine evolution reaction and the water oxidation reaction.^127^ Liu et al.^126^ employed a solid oxide electrolyzer to directly split untreated seawater, operating at a constant current density of 200 mA/cm^2^ for 420 hours and achieving a hydrogen production rate of 183 mL/min. The results demonstrated the cell’s stability even during long-term operation.

Several studies have explored different energy sources for steam generation in SOE systems, such as geothermal energy, coal-fired power plants, municipal waste incineration, and solar energy.^128^ Sanz-Bermejo et al.^129^ investigated the integration of a solid oxide electrolysis cell unit into a direct steam generation solar tower plant. Several configurations were examined to minimize the integration penalties on the concentrating solar power (CSP) plant and enhance the electrolysis performance. The optimal scenario was identified as operating the electrolyzer at low pressure while utilizing its exhaust stream heat to preheat the solar plant feedwater. A recent study by Liu et al.^130^ developed an SOE system capable of adapting to solar radiation fluctuations by introducing a thermal storage module. The system outperformed a conventional solar hydrogen setup, achieving a 9.8% improvement in efficiency. Yang et al.^131^ developed four machine learning models (RF, DNN, SVR, and XGBoost) to predict Ohmic resistance, current density, and H_2_ production rate of SOEC processes at different operating conditions. The results showed that the XGBoost model coupled with the genetic algorithm (GA) demonstrated the highest H_2_ production rate with the lowest current density. However, experimental validation is required.

SOE operates at high temperatures, which enhances cell efficiency; however, these conditions also accelerate electrolyte degradation, thereby reducing the system’s lifespan. Furthermore, current SOE demonstration units are still limited to the kilowatt scale, restricting their feasibility for large-scale applications.^118^

#### 3.1.4 Anion Exchange Membrane (AEM)

Anion-exchange membrane water electrolysis (AEM) was developed to address the limitations of both PEM and AWE systems. It overcomes the high capital cost of PEM, which arises from the use of noble-metal-based electrodes, and the low current density and poor gas purity typical of AWE.^132^ It is a newly established technology and less explored compared to PEM and AWE.^116^ In the AEM cell (Figure 5d), the electrode chambers are separated by a thin, dense, nonporous polymer membrane, while charge transfer occurs via hydroxide ions, as in AWE. The high-pH environment minimizes corrosion, allowing steel to replace titanium in the porous transport layers and nickel to substitute for platinum metals in the electrodes.^133^

In AEM electrolysis, hydrogen is generated at the cathode through the hydrogen evolution reaction driven by electrons, while oxygen forms at the anode via the oxygen evolution reaction driven by the migrated OH^–^.^132,134^ . Park et al.^135^ investigated key factors influencing AEM performance. By optimizing membrane-electrode assembly fabrication, operating conditions, and introducing a new membrane-ionomer combination, they achieved higher current density through reduced ohmic, charge-transfer, and mass transport resistances. Zheng et al.^136^ developed advanced AEM with a high conductivity and durability, along with selected ionomers, catalysts, and porous transport layers. The optimized system achieved stable operation at 10 A·cm^–2^ with a lifetime improvement of five orders of magnitude over current benchmarks, demonstrating the strong potential of high current density AEM electrolysis. Jiang et al.^137^ synthesized a poly (biphenyl alkylene) (PBPA) anion-exchange membrane for use in a highly efficient and durable AEM water electrolyzer. They investigated the membrane electrode assembly (MEA), fabrication parameters, and electrolysis operating conditions. The PBPA-based AEM exhibited outstanding performance and long-term stability, demonstrating strong potential for industrial-scale hydrogen production. Recent studies on water electrolysis technologies are listed in Table 3. Table 4 compares different water electrolysis technologies, and Table 5 lists some commercial green hydrogen production.

**Table 3:** Recent Studies on Water Electrolysis Technologies for Hydrogen Production

technology	study objective	electrolyte/Membrane	cathode	anode	main findings	references
AWE	to investigate utilizing 3D-printing cathodes coated with electrodeposition for hydrogen production	KOH (≥85%)	Ni-NiZnFe_4_O_4_ coated	stainless steel	- the triangular Ni-NiZnFe_4_O4 coated electrode exhibited the higher current density, emphasizing that surface geometry affects electrode performance	^138^
					- the highest energy efficiency was 14.34%	
AWE	exploring hydrogen production using a Ni-Co-nano-graphene thin film cathode by electrodeposition	KOH (25%)	Ni-Co-Nano-graphene	Nickel	adding nano-graphene to the cathode decreased power consumption by about 8.1% compared to the pure nickel cathode	^139^
AWE	investigating the improving of electrolyte absorption in AWE by controlling the porosity of polybenzimidazole membranes	KOH (95%)	Nickel foam	Nickel foam	- the porosity improved the electrolyte absorption, and reduced the areal resistance	^140^
					- the proposed membrane showed excellent performance with the highest reported current density of 2000 mA/cm^2^ for 700 hr	
PEM/AWE	to propose hybrid proton exchange membrane and alkaline electrolyzers utilizing wind and photovoltaic power, while using a novel scheduling method to optimize the operation	KOH (20–40%) for AWE			- the proposed system yielded 3,18,516.76 kg H_2_ in three months	^141^
					- the proposed system reduced switch action times, which improved the economic performance	
AWE	to explore the effect of the electrode-diaphragm gap on H_2_ crossover employing Zirfon UTP Perl 500 and UTP 220 diaphragms	KOH (12 wt %)	Nickel	Nickel	- zero-gap configuration showed high H_2_ crossover driven by supersaturation at diaphragm-electrolyte interface. Whereas a finite gap exhibited lower crossover	^142^
					- introducing a narrow cathode-gap improved gas purity by reducing crossover	
PEM	proposing and validating of a 3D, 2-phase PEM electrolysis comprehensive model coupled with electrochemical reactions, 2-phase flow, and heat transfer models to explore how 2-phase flow affects cell performance	Nafion N117 membrane	Pt supported on carbon catalyst	Ir and IrO_2_ catalyst	bubble coverage on catalyst active sites is the main reason for mass transfer decrease, which can be mitigated by adjusting membrane wettability to improve gas discharge	^143^
Hybrid PV-PV/T PEM	proposing a hybrid photovoltaic/thermal driven PEM system with thermal management for H_2_ production, and investigating the effect of varying solar conditions on the cell performance	Nafion membrane	platinum/titanium (Pt/T)	iridium oxide (IrOx)	- the maximum obtained solar-to-H_2_ efficiency was 7.6% and the average energy efficiency was 75.5%, emphasizing the role of thermal management in improving efficiency and reducing energy consumption	^144^
					- the higher feedwater temperatures reduced the required voltage and enhanced energy efficiency	
SOE	to explore the effect of cathode thickness on the performance of SOEC for H_2_ production	La_0·9_ Sr_0.1_G a_0.8_Mg_0·2_O_3_ (LSGM)	NiFe_2_O_4_ (NFO)	Ba_0·4_La _0·6_ CoO_3_ (BLC)	- the best performance of the SOEC was obtained at 30 μm-NFO cathode thickness	^145^
SOE	to develop a numerical model combining thermal and electrochemical approaches to investigate H_2_ production by SOEC at high temperatures. The developed model was validated against literature data	Yttrium-stabilized zirconia (YSZ)	Yttrium-stabilized zirconia (YSZ) mixed with strontium-doped lanthanum manganate (LSM)	YSZ doped with Nickel	- ohmic polarization is the main reason for performance loss	^146^
					- sweep gas, for O_2_ removal, and a high H_2_O to H_2_ ratio enhanced the cell efficiency	
					- Operating under low current requires an additional heat source	
AEM	to investigate the influence of non-metallic elements (Ni-P, Ni-NC, Ni-NC + Ni-P, and Ni-S/Ni(OH)_2_) on nickel-based electrocatalysts AEM	fumatech FAA-3-50	platinum	Nickel-based anode on porous stainless steel with non-metallic elements (S, P)	- Ni-S/Ni(OH)_2_ outperformed the other tested catalyst materials owing to its high surface area	^147^
					- stability test over a 10-h proved the durability of the anode, with low degradation rate.	
AEM	to investigate membranes synthesized from poly (terphenyl piperidonium) copolymers with a heteroatom-containing π-conjugated structure (dibenzothiophene and dibenzofuran) on AEM water electrolysis performance	poly (terphenyl-co- dibenzothiophene piperidonium) and poly (terphenyl-co-dibenzofuran piperidonium)/ KOH solution	NiCoS	NiFe	dibenzothiophene membrane showed better thermal, chemical stability, and tensile strength	^148^
AEM	exploring quaternary ammonium-type (Cn-PFCO (n = 2, 5)) membrane synthesized from perfluorosulfonic acid resins with carbonyl side chains	Cn-PFCO and C_2_-FPGCI membranes	ink consisted of 50 wt% Pt/C isopropanol, deionized water and C_2_-PFCO solution	ink consisted of IrO_2_, isopropyl alcohol, deionized water and C_2_-PFCO solution	- C_5_-PFCO showed excellent hydroxide conductivity of 113.4 mS cm^–1^ at 80 °C	^149^
					- the presence of carbonyl groups contributed to enhancing the stability by producing anions with hydroxyl groups which reduce the potential for SN2 attack	

**Table 4:** Comparison between Different Water Electrolysis Technologies^4,150,151^

**parameters**	**AWE**	**PEM**	**AEM**	**SOE**
**operating temp. (°C)**	70–90	50–80	40–60	700–850
**operating pressure (bar)**	1–30	<50	<35	1
**H**_**2**_ **purity**	99.9–99.9998%	99.9–99.9999%	99.9–99.999%	99.9%
**voltage range (V)**	1.4–3	1.4–2.3	1.4–2.0	1.0–1.5
**current density (A/cm** ^**2**^ **)**	0.2–0.8	1–2	0.2–2	0.3–1
**efficiency**	50–78%	50–83%	57–59%	∼90% (lab)
**electrolyser lifetime (h)**	60,000	50,000–80,000	>30,000	<20,000
**investment costs in USD/kW**	920–1725	1610–2668	unknown	>2300
**development status**	mature	commercialized	R&D	R&D

**Table 5:** Commercial-Scale Green Hydrogen Projects That Utilize Renewable Energy for Water Electrolysis^152^

**plant location**	**power source**	**electrolyzer capacity (MW)**	**status (as of 2025)**
**Ningxia, China**	solar	150	ongoing
**Kuqa, China**	solar	260	ongoing
**Puertollano, Spain**	solar	20	ongoing
**Hobro, Denmark**	wind	1.2	completed
**Nouadhibou/Inchiri, Mauritania**	solar, wind	400	planned
**Duqm, Oman**	solar, wind	500	planned
**Heide, Germany**	wind	500	planned

### 3.2 Water Electrolysis Mechanism

The hydrogen evolution reaction (HER), which is the cathodic half-reaction of water electrolysis, plays a crucial role in sustainable green hydrogen production and has been widely studied. The HER can follow three primary reaction pathways: Volmer, Heyrovsky, and Tafel mechanisms. In neutral media, this reaction involves a series of electrochemical steps.^153,154^ The HER is initiated by the Volmer step in which an adsorbed hydrogen (H*) forms on the catalyst surface. Subsequently, molecular hydrogen is generated either through the electrochemical Heyrovsky step or the chemical Tafel step.^155^ The binding strength between the catalyst surface and the hydrogen intermediate is a critical factor governing the HER kinetics. According to the Sabatier principle, the adsorption of hydrogen should be neither too weak nor too strong, thereby facilitating both the formation of the adsorbed hydrogen and the subsequent desorption of H_2_.^156^ The three primary HER steps are as follows:

Volmer step:

$$H_{2}O+e+*\to H^{*}+{\mathrm{OH}}^{-}$$

Tafel step:

$$2H^{*}\to H_{2}+2^{*}$$

Heyrovsky step:

$$H_{2}O+H^{*}+e\to H_{2}+{\mathrm{OH}}^{-}+*$$

Alkaline HER electrocatalysts can be grouped into three main types: (i) noble metals and their alloys like Pt, Pd, Ir, Ru, and Ag; (ii) transition metal-based catalysts and their heterostructures, including Fe, Co, Ni, Mn, Cu, Mo, and W; and (iii) metal-free catalysts made from nonmetal elements such as B, C, N, P, and S.^157^

#### 3.2.1 Noble-Metal-Based HER Electrocatalysts

Platinum (Pt) and Pt-based catalysts remain the benchmark HER electrocatalysts because of their exceptional catalytic activity and are widely employed in commercial Pt/C catalysts. The superior HER activity of Pt arises from its optimal adsorption strength for reaction intermediates, which effectively balances adsorption and desorption, and whose Gibbs free energy of H_2_ adsorption is close to zero.^157,158^ However, their widespread industrial application is constrained by high cost and limited stability. Consequently, recent research has focused on reducing Pt loading while enhancing catalyst durability without compromising HER performance.^153^ For instance, Liu et al.^159^ developed a low-Pt-loading single-atom catalyst by anchoring Pt atoms onto hydrogenated MoO_3_ (H_x_MoO_3_) supported on carbon cloth. The resulting Pt/H_x_MoO_3_@CC catalyst exhibited excellent HER activity, requiring an overpotential of only 53 mV at 10 mA cm^–2^, demonstrating that minimizing Pt loading while maintaining high catalytic performance is a promising strategy for practical water electrolysis. Similarly, Wang et al.^160^ developed a low Pt-loading TiO_2_-supported catalyst (TiO_2_-Pt) for the hydrogen evolution reaction (HER) in PEM. The strong interaction between TiO_2_ and Pt promoted electron transfer and facilitated the Heyrovsky step. When integrated into a PEM, the catalyst achieved a cell voltage of 1.65 V at 1 A cm^–2^ and maintained stable operation for 300 h.

#### 3.2.2 Non-Noble Metal-Based HER Electrocatalysts

Transition-metal catalysts such as Fe, Co, and Ni are extensively studied as affordable alternatives to noble metals because their partially filled d orbitals enable electron transfer in the HER. However, their lower inherent catalytic activity, especially in neutral environments, restricts their efficiency, emphasizing the importance of effective modification techniques to boost their activity and practical use.^158,161^ Zhao et al.^162^ synthesized a honeycomb-structured bimetallic phosphide catalyst (MoP/MoNiP@NPC-800) using a one-step pyrolysis process. This catalyst demonstrated outstanding HER performance in both alkaline and acidic conditions, with low overpotentials of 50.4 mV in 1.0 M KOH and 72.6 mV in 0.5 M H_2_SO_4_ at 10 mA/cm^2^, and maintained stability for around 120 hours. The improved activity resulted from the synergistic effects of MoP and MoNiP active sites and the honeycomb porous structure, which increased the active site exposure, enhanced mass transfer, and prevented catalyst agglomeration and corrosion. Jiang et al.^163^ prepared a bimetallic NiCoP catalyst grown on carbon cloth (NiCoP/CC) using a one-step potentiostatic electrodeposition process. Compared to monometallic NiP and CoP catalysts, NiCoP/CC showed better HER performance under both acidic and alkaline conditions, maintaining stability at 10 and 100 mA cm^–2^ over time. This improved activity results from the synergistic interaction between Ni and Co, which enhances the electronic structure, and the multi-meso-porous architecture that offers high surface area, better electrical conductivity, and numerous active sites. This research highlights the potential of NiCoP/CC as a cost-effective, efficient, non-noble-metal catalyst suitable for large-scale hydrogen production.

#### 3.2.3 Electrocatalyst Deactivation Mechanisms

Electrocatalysts are suspected to deactivate chemically via dissolution, crystal transformation, and passivation. Catalyst dissolution is a major cause of electrocatalyst deactivation during water electrolysis. Under prolonged operation, both the catalyst and its support may undergo chemical or electrochemical dissolution, leading to reduced activity and stability. Furthermore, dissolved species can redeposit on the electrode surface, blocking active sites, increasing voltage losses, and weakening interactions between the catalyst and reactants, thereby accelerating performance degradation.^164^ Particle agglomeration happens when catalyst nanoparticles move across the support surface and merge into larger clusters, which decreases the number of active sites available.^165^ Smiljanić et al.^166^ reported that weak interactions between Pt nanoparticles and carbon supports can lead to catalyst degradation via particle agglomeration during HER. Strengthening the strong metal-support interaction (SMSI) is an effective strategy to improve both the activity and long-term stability of Pt-based electrocatalysts. Therefore, they developed a Pt/TiON_x_ nanocomposite by supporting Pt nanoparticles on conductive titanium oxynitride dispersed over reduced graphene oxide nanoribbons, which outperformed the commercial Pt/C catalyst. The improved performance was attributed to the strong metal–support interaction (SMSI) between Pt and TiON_x_, which stabilized the Pt nanoparticles and optimized hydrogen adsorption, as confirmed by XPS and density functional theory (DFT) calculations. Catalyst detachment is another important physical degradation mechanism that is primarily caused by the weakening of the interaction between the catalyst layer and the support material, leading to catalyst loss and reduced electrocatalytic performance.^157^ Paciok et al.^167^ investigated the degradation of carbon-supported Pt catalysts under PEM water electrolysis. They found that hydrogen evolution promoted the migration and detachment of Pt nanoparticles from the carbon support, resulting in particle agglomeration and catalyst deactivation. The degradation was attributed to weakened van der Waals interactions caused by high hydrogen coverage on Pt, highlighting that strengthening Pt-support adhesion is an effective strategy for improving catalyst durability.

### 3.3 Water Photocatalytic and Photoelectrochemical Splitting

Photocatalytic and photoelectrochemical (PEC) water splitting are the two primary approaches for directly converting sunlight into hydrogen.^168^ In the particulate photocatalyst (PC), as shown in Figure 6a, water splits into hydrogen and oxygen while the photocatalyst is suspended in an aqueous medium under light irradiation.^169^ Light excitation generates electron-hole pairs. These charge carriers then separate and migrate to the surface, where they are captured by reduction and oxidation co-catalysts. Once trapped, the electrons and holes participate in the catalytic reactions that reduce and oxidize water, producing hydrogen and oxygen. In particulate photocatalyst systems, which are the simplest and most cost-effective approach for potentially large-scale solar hydrogen production, one major drawback is the need for efficient H_2_/O_2_ gas separation and the requirement for an enclosed reaction setup.^170^ The mixed H_2_/O_2_ gases generated during photocatalytic water splitting must be safely separated, which can be achieved through molecular sieving, taking advantage of the difference in their kinetic diameters (H_2_: 2.9 Å; O_2_: 3.5 Å). This additional separation step, however, increases system complexity and poses safety and engineering challenges for scale-up.^170^ Nishiyama et al.^171^ successfully developed a 100-m^2^ array of panel reactors over several months with hydrogen recovery using a commercial polyimide membrane. This demonstrates the possibility of safe, large-scale photocatalytic water splitting, gas collection, and separation.

**Figure 6:**
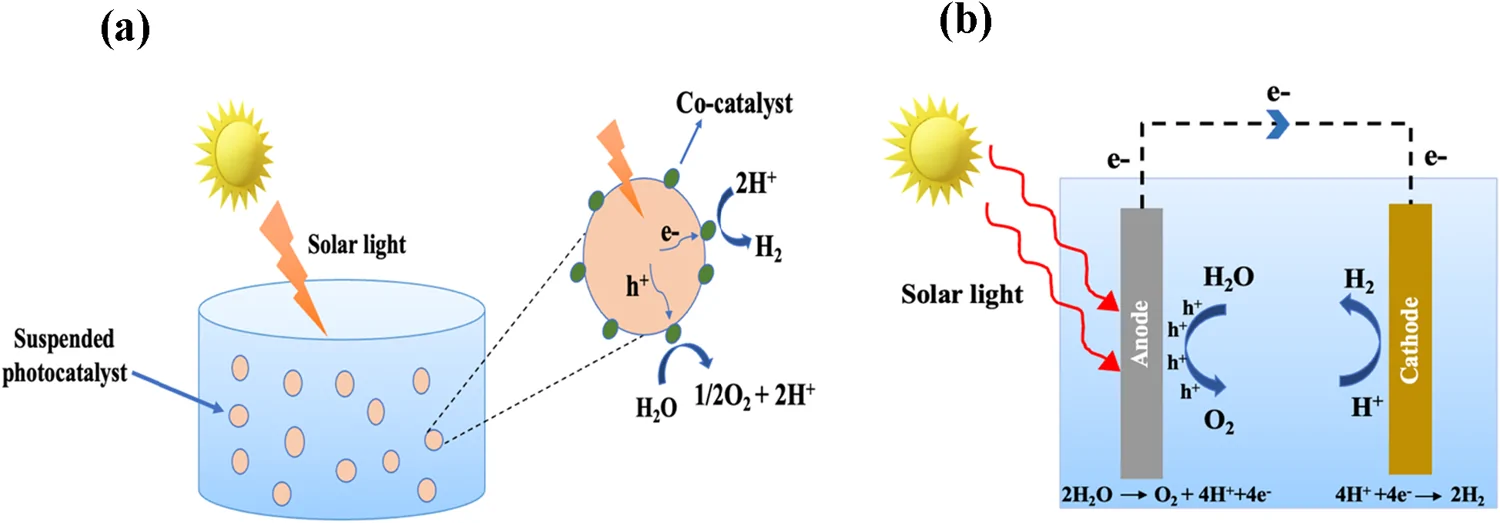
Schematics for (a) photocatalytic water splitting and (b) photoelectrochemical water splitting.

Another key limitation of photocatalysis is the rapid recombination of photogenerated electrons and holes, which occurs much faster than the desired electrochemical reactions and significantly reduces overall efficiency.^172^ Charge recombination can be minimized by using catalysts with a higher specific surface area and smaller particle size. As particle size decreases, the travel distance required for photogenerated charge carriers becomes shorter, which further promotes efficient charge separation.^173^ Jiang et al.^174^ enhanced the photocatalytic activity of TiO_2_ for hydrogen production by incorporating it into microporous ZSM-5 and mesoporous SBA-15 using a sol-gel impregnation method. The improvement is mainly due to the high surface areas of these supports, which help stabilize well-dispersed TiO_2_ nanocrystallites and promote more efficient photocatalysis. He^175^ developed a flower cluster ZnO/ZnS/CdS three-phase composite photocatalyst designed to promote rapid separation of photogenerated electrons and holes, while also enhancing the catalyst’s stability during photocatalytic hydrogen production.

In PEC water splitting, a semiconductor photocatalyst is deposited onto an electronic substrate to form photoanodes or photocathodes, and electron transfer occurs at the semiconductor-electrolyte interface.^170^ As shown in Figure 6b, in PEC water-splitting, the semiconductor electrode submerged in water absorbs the solar light, which excites the electrons and generates electron-hole pairs, these charge carriers then separate and migrate to the semiconductor-liquid interface, the electrons transfer over the internal circuit to the cathode for hydrogen formation.^176^

The PEC water-splitting process has emerged as a promising method for producing green hydrogen, with advantages such as zero hazardous gas emissions and low operational costs. PEC has attracted significant attention as a potential alternative to rapidly depleting fossil fuels.^176^ Catalysts such as metal oxides (e.g., titanium dioxide) and metal chalcogenides (e.g., cadmium sulfide) are common in PEC.^177^ BiVO_4_ and Ta_3_N_5_ are widely used in PEC systems because their broad light absorption range enables higher solar-to-hydrogen conversion efficiency.^170^ Sudi et al.^178^ demonstrated that coupling metalloporphyrins with semiconductor materials is an effective strategy for enhancing photocatalytic performance. They investigated three BiVO_4_-based composite catalysts incorporating Co-, Ni-, and Cu-metalloporphyrins. It was found that the Co-porphyrin catalyst exhibited the highest activity, which was attributed to improved charge transfer and reduced electron-hole recombination. Xie et al.^179^ developed a novel homogeneous BVO/BiVO_4_ photoanode composed of BiVO_4_ nanodots with exposed (011) facets and further improved its activity by photodepositing a NiFeOx electrocatalyst layer. The optimized NiFeOx/BVO/BiVO_4_ photoanode delivered a current density of 6.40 mA cm^–2^ at 1.23 V, about 4.7 times higher than that of pristine BiVO_4_, offering a promising approach for enhancing the PEC water-splitting performance of BiVO_4_-based photoanodes.

### 3.4 Water Thermochemical Splitting

Thermolysis involves the direct dissociation of water at extremely high temperatures, typically above 3000 K at 1 bar. Developing materials capable of withstanding such severe temperatures remains a significant challenge. In addition, thermolysis faces safety concerns due to the generation of an explosive hydrogen and oxygen mixture, requiring complex separation processes. These limitations have restricted the progress of direct solar thermal water splitting. Thus, research efforts have shifted toward multi-step thermochemical water-splitting cycles that utilize redox materials and operate at relatively lower temperatures.^180,181^ Two and three-step water-splitting cycles are the most suitable processes for integration with solar energy. As shown in Figure 7,^182^ in a typical two-step cycle, the process begins with the endothermic reduction of a metal oxide at a high temperature (above 1300 °C). The reduced oxide then reacts with water at a lower temperature to produce hydrogen, reproducing the original metal oxide.^183,184^

**Figure 7:**
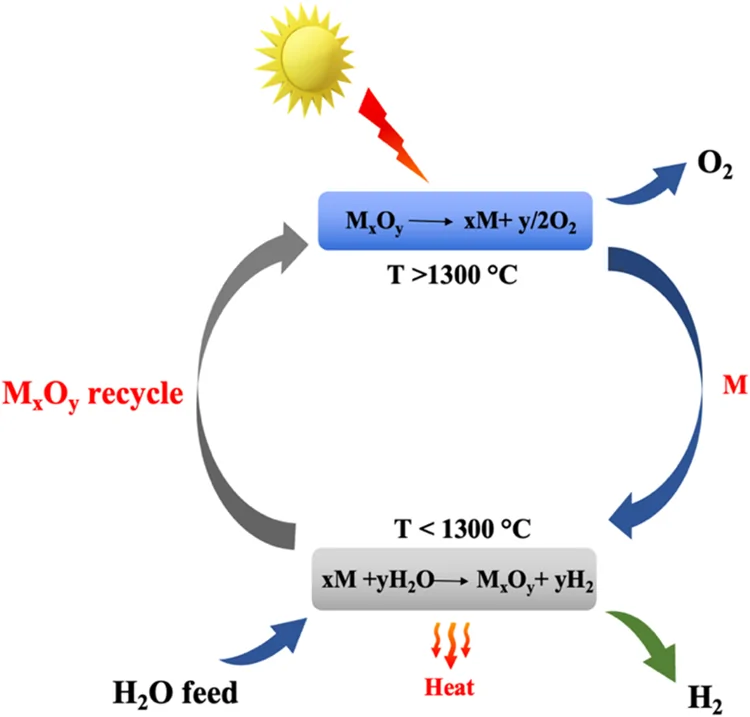
Thermochemical water splitting steps.

Multiple two-step water-splitting cycles based on volatile and non-volatile metal oxides redox pair reactions, such as ZnO/Zn, Fe_3_O_4_/Fe, SnO_2_/SnO, CeO_2_/Ce_2_O_3_, Mn_2_O_3_/MnO, Co_3_O_4_/CoO, CdO/Cd, GeO_2_/GeO have been investigated. Zn, Fe, and Ce are the most widely investigated metals.^185^ Koepf et al.^186^ were able to demonstrate the first 100 kW_th_ pilot-plant reactor using ZnO as the metal oxide in a two-step water-splitting cycle. The system operated for 100 hours, converting more than 4000 kW/m^2^ of concentrated solar radiation into heat, and achieved a solar-to-chemical efficiency of 3%; however, separation of the products zinc and oxygen was the main challenge. Ceria (CeO_2_) is considered a high-performance redox material for thermochemical cycles, owing to its excellent crystallographic stability and fast water-splitting kinetics. However, it requires high temperatures to achieve better hydrogen yield due to its limited oxygen vacancies.^187^ Thus, it has been proposed that doping systems, such as CeO_2_/m-ZrO_2_ and Fe_3_O_4_/m-ZrO_2_, have the potential to lower the operating temperature of thermochemical redox reactions. However, it reduces the hydrogen yield.^185^ Arifin et al.^188^ studied the effect of adding zirconium as a dopant into the ceria lattice. It was found that O_2_ and H_2_ production increased by approximately 30 and 11%, respectively. In contrast, adding Pr and Gd to the binary Zr-ceria mixed oxide reduced H_2_ production. The major drawbacks of two-step thermochemical cycles are their lower efficiency compared to electrolysis and the need for materials that can sustain the high operating temperatures.^189^ Previous research suggests that thermochemical water-splitting cycles integrated with nuclear, geothermal, wind, or solar energy sources offer the lowest hydrogen production costs, which are in the ranges of 1.28–15.56/kg H_2_ for Cu-Cl cycle, 6.14–11.60 USD/kg H_2_ for sulfur-iodine cycle, and 3.97–6.01 USD/kg H_2_ for hybrid sulfur cycle.^190^ Table 6 summarizes the latest studies on water photoelectrochemical and thermal splitting for hydrogen production.

**Table 6:** Recent Studies on Water Photoelectrochemical and Thermal Splitting for Hydrogen Production

technology	study objective	cathode	anode	main findings	references
PEC	to explore the impact of a new design of mesh photocathode on hydrogen production rates and the efficiency under varying light angles due to the sun movement	stainless steel with cuprous oxide (Cu_2_O) coating	stainless steel Titanium dioxide (TiO_2_) coating	- the maximum H_2_ production rate is 15.83 μg/s for vertical light positions	^191^
				- the maximum exergy and energy efficiencies of 2.19 and 3.20% were achieved under vertical light conditions	
PEC	to synthesize pure iron tungstate nanoparticles (p-FeWO_4_) and applying as a co-catalyst onto porous anodic WO_3_	Pt mesh	FeWO_4_/WO_3_	- the thin FeWO_4_ layer contributed to sufficient light exposure of the underlying WO_3_	^192^
				- the fabricated photoanode suppressed charge recombination, thus achieving a stable photocurrent density 1.5 times higher than that of pristine WO_3_	
PEC	to propose Chromium (Cr) doping BiVO_4_ to enhance the PEC properties for H_2_ production	Pt	Cr doped BiVO_4_	3 %Cr photoanode doping showed long-term stability, while 2 %Cr achieved the highest instantaneous current, emphasizing Cr as an effective dopant for improving BiVO_4_ performance	^193^
thermal water splitting	to propose a novel hydrogen production system using the three-step copper chlorine (Cu-Cl) cycle without an electrolysis step, and investigating parameters such as temperature and steam/copper ratio on hydrogen production			- H_2_ concentration increased as the temperature and time increased	^194^
				- the maximum H_2_ production obtained at a steam-to-cupper ratio of 55 and a temperature of 400 °C	
thermal water splitting	to investigate thermochemical water splitting for H_2_ production based on perovskite La_0.8_Sr_0.2_CoO_3‑δ_ under different temperature conditions.			the results revealed that at thermal reduction temperatures >1000 °C, H_2_ production decreased at each cycle. however, at lower temperatures the material exhibited more stability	^195^
thermal water splitting	to explore hydrogen production by a two-stage thermochemical water-splitting cerium oxide medium reactor powered by a parabolic dish receiver and a photovoltaic heater, and proposing a numerical simulation and experimental validation			- PV heater performed better than solar concentration.	^196^
				- the increase in N_2_ flow rate improved the heat transfer and increased H_2_ production during the oxidation stage	
				- the maximum H_2_ production was 2000 mol of H_2_ per gram of ceria	

## 4 Comparison between Different Methods

Table 7 provides a comparative overview of the principal hydrogen production technologies, covering their technology readiness level (TRL), hydrogen yield, operating conditions, advantages, disadvantages, and key challenges. This comparison facilitates the evaluation of each technology’s maturity, performance, and suitability for large-scale implementation.

**Table 7:** Comparison between Hydrogen Production Technologies^4,84,151,197–207^

technology	TRL	H_2_ yield (g H_2_/kg feedstock)	operating conditions	advantages	disadvantage	challenges
AWE	9		70–90 °C, 1–30 bar	mature and well-established technology	• corrosive alkaline electrolyte	• increase current density while reducing overpotential through improved electrocatalysts, electrode materials, and cell design
				• uses non-precious metal (PGM-free) electrocatalysts	• gas crossover through porous diaphragms	• develop zero-gap cell configurations to minimize ohmic resistance and enhance efficiency
				• relatively low capital cost	• lower hydrogen purity	
				• excellent long-term stability	• limited current density	
				• suitable for MW-scale operation	• slow start-up and system response	
PEM	9		50–80 °C, <50 bar	• commercially mature technology	• requires an acidic PFSA membrane	• minimize dependence on platinum-group metals
				• compact stack configuration	• depends on platinum-group-metal catalysts	• develop high-performance PGM-free electrocatalysts
				• capable of operating at high pressure and under differential pressure conditions	• high cost of membranes and stack components.	• improve membrane durability and efficiency
				• achieves the highest current densities among electrolysis technologies.	• long-term durability at MW scale still requires validation	• reduce membrane thickness to improve performance
				• produces high-purity hydrogen		
				• rapid start-up and fast dynamic response		
				• well suited for fluctuating renewable power sources		
AEM	6		40–60 °C, <35 bar	• combines key features of AWE and PEM technologies	• relatively low ionic conductivity	• improve membrane durability and reduce polymer degradation
				• operates with dilute alkaline electrolytes	• limited membrane stability	• develop testing methods that accurately replicate practical AEM operating conditions
				• enables the use of PGM-free electrocatalysts	• long-term lifetime has not yet been fully demonstrated	
				• suitable for variable renewable energy operation	• technology is still under development	
SOEC	6		700–850 °C, 1 bar	• lower electrical energy consumption	• limited long-term stability due to electrochemical and mechanical degradation	• improve control of electrochemical degradation
				• high conversion efficiency	• shorter durability and operational lifetime	• enhance thermo-mechanical stability of cell components
				• operates at elevated temperature and pressure	• still under development.	• develop materials resistant to high temperatures and humidity for prolonged operation
				• does not require noble-metal catalysts		
pyrolysis	3–4 (lab scale)	25–65 g H_2_/kg feedstock	300–700 °C, inert conditions	• based on established industrial process design	• char and tar generation.	• advancement of TRL beyond laboratory-scale hydrogen production
				• oxygen is not required	• catalyst regeneration requirements	• development of efficient gas-cleaning technologies to enhance hydrogen quality and yield
					• post-treatment of product gases is necessary	
					• hydrogen composition varies due to biomass complexity and heterogeneity	
					• relatively low hydrogen yield	
					• high reactor cost	
gasification	5–6	40–190 g H_2_/kg feedstock	500–1200 °C, steam or air gasifying agent, atmospheric or pressurized conditions	• established industrial process	• elevated operating temperatures	• improved tar removal and gas purification to enhance hydrogen quality and yield
				• high biomass conversion efficiency	• tar and char formation leading to catalyst deactivation	• catalyst optimization
				• produces comparatively high hydrogen yields among biomass conversion routes	• catalyst regeneration is required	
				• steam gasification avoids the need for costly oxygen sources	• hydrogen composition varies with biomass characteristics	
					• feedstock pretreatment is required	
					• product gas requires post-treatment because of fluctuations in gas composition	
					• expensive reactor systems	
steam reforming		40–130 g H_2_/kg feedstock	600–900 °C, 10–30 bar, Ni based catalysts. 10.5 kg CO_2_ per kg of H_2_	• established industrial process design	• high operating temperatures	• improving the cost-effectiveness of steam reforming for green hydrogen production by utilizing readily available intermediate products
				• no oxygen requirement	• Catalyst regeneration requirements	• maintaining competitiveness with alternative technologies that also utilize intermediate feedstocks
				• high conversion efficiency	• high energy demand	
				• one of the highest hydrogen yields among other technologies.		
				• well-established and widely implemented technology		
dark fermentation	5–6	1–75 mL H_2_/g	30–55 °C (mesophilic and thermophilic), 5.0–6.5 pH	• operates under low temperature and pressure	• low hydrogen production rate and yield	• advances in genetic engineering of microorganisms
				• compatible with a wide variety of carbon sources and substrates	• high generation of by-products	• optimization of substrate utilization, process conditions, and reactor design
				• does not depend on light	• separation of H_2_ and CO_2_ results in low hydrogen purity in the product gas	
				• no oxygen restriction	• limited substrate conversion efficiency	
				• can utilize algal biomass with high growth rates		
photofermentation	5	12–408 mL H_2_/h	31–36 °C, 6.8–7.5 pH	• operates under mild temperature and pressure	• feedstock pretreatment is required	• further advances in genetic engineering
				• can utilize a wide variety of substrates	• low hydrogen production rate and yield	• optimization of substrate utilization, operating conditions, and reactor design, particularly to address the high surface area requirement
				• photosynthetic bacteria are capable of harvesting a broad spectrum of light	• hydrogenase activity is inhibited by oxygen.	
				• can use the effluent generated from dark fermentation	• requires an external light source	
				• enables nearly complete substrate conversion	• limited light conversion efficiency	
					• control of bacterial growth is required	
PEC	3–5		temperature (45–55 °C), solar illumination (AM 1.5G, 100 mW cm^–^^2^), aqueous or solid electrolyte, 1.4–1.5 V applied bias	• direct conversion of solar energy into hydrogen in a single integrated device	• high hydrogen production cost	• develop low-cost, scalable photoelectrodes
				• low environmental impact	• low long-term durability	• improve STH efficiency and long-term stability (>1000 h)
				• opportunity for thermal management to improve energy conversion efficiency	• limited scalability	• reduce ohmic losses
					• high cost and complexity of photoelectrodes	• optimize thermal and power management
					• low solar-to-hydrogen (STH) efficiency compared with commercial targets	• improve gas separation and large-area device design
						• standardize technoeconomic and environmental evaluation methods
MEC	4–6	1 L H_2_/day/m^2^	ambient temperature (20–35 °C), near-neutral pH (6.5–7.5), anaerobic conditions, applied voltage 0.2–1.0 V, wastewater or organic substrates	• simultaneous hydrogen production and wastewater treatment	• low current density at pilot scale	• improve current density and hydrogen production rate
				• low energy requirement compared with conventional water electrolysis	• hydrogen losses due to leakage and methanogenesis	• reduce electrode and reactor costs
				• utilizes wastewater instead of purified water	• high internal resistance in large reactors	• improve hydrogen recovery and suppress methanogenesis
				• potential for energy-positive wastewater treatment	• electrode and membrane costs remain high	• optimize reactor design and scale-up strategies
				• can become economically viable under favorable market conditions	• limited long-term operational data and commercial deployment	• standardize performance and technoeconomic reporting
						• enhance long-term operational stability and durability

## 5 Technoeconomic Analysis and Life Cycle Assessment

Technoeconomic assessment (TEA) is a structured method for appraising hydrogen production methods, focusing on essential economic and technical metrics such as the levelized cost of hydrogen (LCOH), capital expenditure (CAPEX), operational expenditure (OPEX), system efficiency, and stack lifespan.^208^ Aminaho et al.^209^ provided a comparative technoeconomic analysis (TEA) of alkaline water electrolysis (AWE), proton exchange membrane (PEM), solid oxide electrolysis cells (SOEC), and anion exchange membrane (AEM) technologies, with costs normalized to 2024 USD to ensure consistent comparison, as listed in Table 8. A total of 47 studies satisfied the inclusion criteria and were retained for the final synthesis, with the selection process illustrated by using the PRISMA 2020 flow diagram. Mehmeti et al.^210^ performed TEA for hydrogen production methods and estimated the resource requirements as listed in Table 9.

**Table 8:** Comparative TEA for Water Electrolysis Technologies^209^

**parameter**	**AWE**	**PEM**	**SOEC**	**AEM**
**CAPEX (USD/kW)**	1816	2147	∼3000+ (estimated)	<1500 (projected)
**LCOH (USD/kg H**_**2**_ **)**	2.85–6.52	2.34–7.52	1.5–5.0	2.5–5.0
**stack lifetime (h)**	80,000	40,000–60,000	20,000–30,000	2000–8000
**efficiency (%)**	60–70	60–70	70–90	50–65
**best-fit application**	geothermal	PV-wind hybrid	concentrated solar power (CSP), organic rankine cycles (ORC), waste heat systems	decentralized rural, low-cost systems

**Table 9:** Resources Required to Produce 1 kg of H_2_ for Different Technologies^210^

**resources**	**steam methane reforming**	**biomass gasification**	**PEM**	**SOEC**	**dark fermentation and MEC without energy recovery**	**dark fermentation and MEC with energy recovery**	**dark fermentation and MEC with H**_**2**_ **recovery**
**feedstock**	natural gas (165 MJ/kg H_2_)	corn stover (13.5 kg/kg H_2_)			corn stover (23.0 kg/kg H_2_)	corn stover (23.0 kg/kg H_2_)	corn stover (23.0 kg/kg H_2_)
**electricity (kWh/kg H**_**2**_ **)**	1.11	0.98	54.6	36.14	21.6	6.03	21.6
**water (kg/kg H**_**2**_ **)**	21.86	305.5	18.04	9.10	104.22	104.22	104.22

Pinaud et al.^211^ conducted one of the initial extensive technoeconomic analyses (TEAs) of large-scale photoelectrochemical (PEC) hydrogen generation. Their goal was to identify the conditions that would make PEC water-splitting financially competitive with traditional hydrogen production methods. The research examined how capital and operating expenses, solar-to-hydrogen (STH) efficiency, device lifespan, and panel costs affect the levelized cost of hydrogen (LCOH). They found that STH efficiency, device durability, and manufacturing costs are the key factors influencing the economic feasibility of PEC water splitting, with hydrogen production costs estimated to be between USD 4.1 and 10.4 per kilogram of H_2_ under various design scenarios.

Jiang et al.^204^ performed a technoeconomic analysis to assess MEC profitability by comparing revenue from hydrogen sales to electricity costs. Hydrogen production per unit of electricity was influenced by the cell voltage and cathodic hydrogen recovery; lower voltages and higher recovery improved energy efficiency. Under both theoretical and operational conditions, hydrogen output was estimated to range from 0.022 to 0.303 kg of H_2_ per kWh. The study also introduced the energy price ratio (EPR), which depends on the market prices of hydrogen and electricity. Results indicated that MEC profitability rises with higher hydrogen prices and lower electricity costs, suggesting that pilot-scale MECs can be economically feasible in markets with favorable conditions. Aiken et al.^212^ established technoeconomic performance targets for the commercialization of microbial electrolysis cells (MECs) using real-world wastewater treatment data. The analysis revealed that the anode and current collector accounted for approximately 94% of the total material cost, indicating that reducing their cost by ∼90% is essential for financial competitiveness. Furthermore, the model showed that operating at organic loading rates of 800–1400 g COD m^–3^ day^–1^ and extending the bioanode lifetime to 20 years would substantially improve the economic feasibility of MEC technology.

Shahabuddin et al.^213^ reported in their review that biomass gasification achieves about 50% thermal efficiency for hydrogen production. The levelized cost of hydrogen (LCOH) is estimated between USD 2.3 and 5.2 per kilogram for plants processing around 10 MWth and from USD 2.8 to 3.4 per kilogram for larger plants exceeding 250 MWth. For residual-waste-based hydrogen, LCOH likely ranges from USD 1.4 to 4.8 per kilogram, influenced by waste gate fees and plant size. The key barriers include waste pretreatment, syngas conditioning, technological maturity, market development, and policy support. Despite these challenges, it emphasizes the potential of biomass gasification for affordable and low-carbon hydrogen. Salkuyeh et al.^214^ carried out a technoeconomic analysis and a life cycle assessment (LCA) to compare hydrogen production methods using fluidized-bed (FB) and entrained-flow (EF) biomass gasification. The findings indicated that EF gasification had a higher thermal efficiency (56% based on the LHV) than FB gasification (45%). However, FB gasification had a lower minimum hydrogen selling price by about USD 0.3 per kilogram of H_2_. When carbon capture and hydrogen liquefaction were included, the thermal efficiencies of the EF and FB systems decreased to 50 and 41%, respectively. Economic analysis suggested that at a biomass feedstock cost of USD 100 per tonne, biomass gasification could compete with traditional steam methane reforming if supported by a carbon price of at least USD 115 per tonne of CO_2_-equiv or a natural gas price of USD 5 GJ^–1^. Additionally, the life cycle assessment (LCA) showed that biomass-based hydrogen production could result in net-negative greenhouse gas emissions due to the carbon-neutral nature of the biomass feedstock.

Life cycle assessment (LCA) is a standardized method for evaluating the environmental impacts of hydrogen production technologies throughout their entire lifespan, from feedstock collection to hydrogen use, following the ISO 14040 and ISO 14044 standards. LCA helps identify environmental hotspots by measuring resource use and emissions, especially greenhouse gases (GHGs).^215^ Compared with traditional fossil fuel-based hydrogen, biomass-derived hydrogen generally shows significantly lower environmental impacts over its life cycle. Among the reviewed processes, dark fermentation resulted in the lowest GHG emissions (15.1 kg CO_2_-equiv per kg H_2_), while gasification (18.0 kg CO_2_-equiv/kg H_2_) and microbial electrolysis cells (18.8 kg CO_2_-equiv/kg H_2_) had the highest GHG emissions. Comprehensive LCA studies are still limited for many emerging hydrogen technologies, particularly hybrid systems, stressing the importance of integrated environmental, technoeconomic, and sustainability assessments to support their commercialization.^216,217^

Photoelectrochemical (PEC) water splitting has among the lowest environmental impacts of hydrogen production methods.^201^ Sathre et al.^218,219^ found that a PEC system with 10% solar-to-hydrogen (STH) efficiency, a 20-year lifespan, and a 1 GW annual capacity achieved an energy payback time (EPBT) of 8.1 years and an energy return on energy invested (ERoEI) of 1.7. Increasing the STH efficiency to 20% decreased the EPBT to about 3 years. The study highlighted that photoelectrode fabrication, module manufacturing, system operation, and end-of-life disposal are the main environmental impact sources. Additionally, PEC systems generally have lower global warming and acidification impacts compared to steam methane reforming (SMR), photovoltaic-electrolysis (PV-EC), and wind-powered PEM electrolysis.

Thompson et al.^29^ reported the environmental impacts of biomass-based processes such as pyrolysis, gasification, and fermentation. Thermochemical methods can have a wide carbon footprint, ranging from −21.8 to 20.8 kg CO_2_-equiv per kg H_2_, and an acidification potential between 1.77 × 10^–3^ and 30.7 kg SO_2_-equiv per kg H_2_. In contrast, biochemical fermentation shows a lower carbon footprint, between 0.32 and 14.25 kg CO_2_-equiv per kg H_2_, with an acidification potential from 3.2 × 10^–4^ to 5.8 kg SO_2_-equiv per kg H_2_. Ozone depletion for thermochemical routes is relatively low (1.19 × 10^–8^ to 2.98 × 10^–8^ kg R11-equiv per kg H_2_), and it is considered negligible in biochemical fermentation. Overall, biochemical pathways tend to have lower environmental impacts than thermochemical ones, though significant variation exists across different processes mainly depending on the biomass feedstock and operating conditions.

Wei et al.^220^ conducted a detailed LCA of four water electrolysis methods: alkaline (AEL), proton exchange membrane (PEM), anion exchange membrane (AEM), and solid oxide electrolysis (SOE). The study covered the entire lifecycle from manufacturing to operation and end-of-life, following ISO 14040 and ISO 14044 standards. It evaluated four environmental impacts: climate change (CC), human health (HH), ecosystem quality (EQ), and abiotic resources (ASR). The analysis was performed across twelve scenarios using photovoltaic, wind, and hydropower sources. Results indicated that wind-powered AEM had the lowest greenhouse gas emissions (0.008 kg CO_2_-equiv MJ^–1^ H_2_), while hydropower-powered AEL had the highest (0.083 kg CO_2_-equiv MJ^–1^ H_2_). The findings also showed that electricity use is the main environmental impact driver, and factors such as electrolyzer efficiency, stack durability, material choice, and renewable electricity usage are crucial for reducing the life cycle greenhouse gas emissions of green hydrogen. Iyer et al.^221^ conducted a detailed LCA of three water electrolysis methods: alkaline electrolysis (AEC), proton exchange membrane electrolysis (PEM), and solid oxide electrolysis (SOEC), focusing on manufacturing and hydrogen generation. Their evaluation considered energy use and greenhouse gas emissions from various electricity sources, including the U.S. grid, nuclear, solar, and wind. Sensitivity analyses explored the impacts of the capacity factor, platinum-group metal (PGM) allocation, and electricity consumption. Findings showed that electrolyzers powered by low-carbon sources emit between 0.3 and 2.4 kg of CO_2_-equiv per kg of H_2_, which is over 50% less than emissions from steam methane reforming and autothermal reforming with carbon capture and storage. The study emphasized that the electricity source significantly influences environmental outcomes. Table 10 lists recent pilot plants for hydrogen production.

**Table 10:** Recent Pilot Plants for Hydrogen Production

country	technology	feedstock	status	capacity	outcomes	refs
Bangladesh	biomass gasification	wood chips	pilot plant	15 kWh	8.6% (vol %) hydrogen gas and stores it at 55 bar for further use	^222^
UK	biomass gasification	NA	pilot plant	0.5 MW H_2_	commercial grade hydrogen	^223^
China	dark and photofermentation	waste biomass	pilot plant	NA	1 t of H_2_ required approximately 171,530 MJ of energy and resulted in 9.37 t CO_2_-equiv emissions, while the estimated economic payback period was 6.86 years	^224^
Colombia	dark fermentation	pig manure, coffee mucilage, cocoa mucilage	pilot scale	NA	the average hydrogen production rate of 3.64 L H_2_/L-d	^225^
Netherlands	biomass gasification	wood and pruning residues	planned commercial	100 MWth gasification input	∼15,000 t H_2_/year	^226^

## 6 Hydrogen Storage and Transportation

Hydrogen storage can be achieved through either physical methods or material-based approaches, as shown in Figure 8.^227^ Physical storage involves compressing hydrogen at high pressures of 350–700 bar or cooling it to cryogenic temperatures (−253 °C) to enhance its energy density per weight. Conversely, material-based storage uses solid materials that can reversibly absorb or adsorb hydrogen. Each method has its own advantages and drawbacks. Current research aims to address material limitations, technological challenges, and improve overall storage efficiency for large-scale hydrogen use.^228,229^ Compressed gaseous hydrogen is the most widely used and straightforward storage method, commonly employed at hydrogen refueling stations for stationary, transportation, and bulk storage applications.^228^ Hydrogen is typically stored in carbon fiber composite pressure vessels or metal tanks at pressures ranging from 20 to 100 MPa, depending on the required weight and storage capacity. Carbon fiber composite tanks are preferred for lightweight applications, whereas metal pressure vessels are used when higher storage capacity is needed. The primary advantages of compressed hydrogen storage are rapid refueling and high discharge rates; however, its widespread use is limited by large storage space requirements and safety concerns associated with high-pressure operation.^227,230^

**Figure 8:**
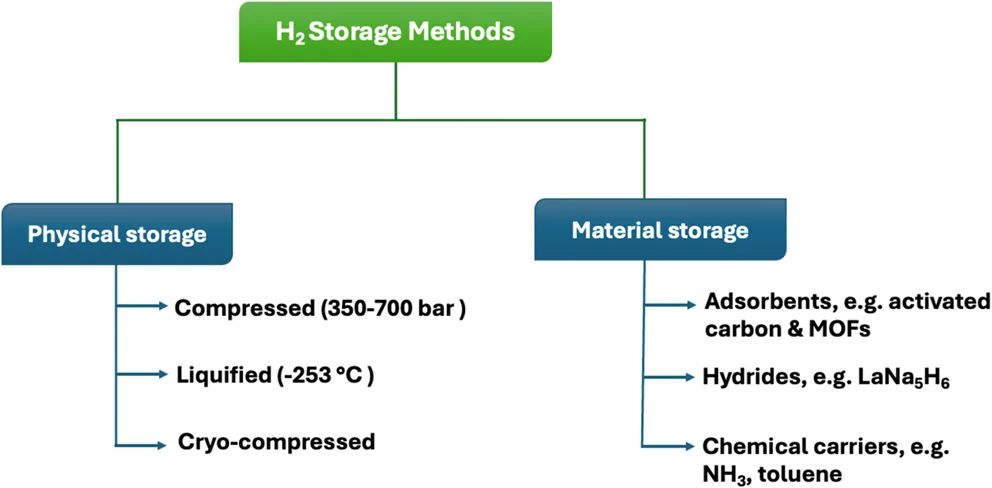
Hydrogen storage methods.

Liquid hydrogen storage offers a higher volumetric energy density than compressed gaseous hydrogen; however, the liquefaction process is energy-intensive. In addition, boil-off losses during storage and transportation remain a major challenge, requiring highly insulated storage tanks to minimize hydrogen evaporation while maintaining high gravimetric and volumetric energy densities.^231^ Liquid hydrogen is typically stored and transported in cryogenic vessels for industrial and medical applications. Although liquefaction at −253 °C provides high volumetric energy density, it is highly energy intensive, consuming approximately 40% of the hydrogen’s energy content, compared with 10–15% for compressed hydrogen storage.^232^ Cryo-compressed hydrogen combines the benefits of compressed gas and liquid hydrogen storage by holding hydrogen at low temperatures under pressure, which enables higher storage densities and reduces boil-off losses. This method can reach hydrogen storage densities of up to 80 g L^–1^, providing better volumetric efficiency than traditional compressed hydrogen storage.^233^

Limitations of compressed and liquid hydrogen storage have heightened interest in materials-based alternatives.^234^ To accelerate their development, the U.S. Hydrogen and Fuel Cell Technologies Office established the Hydrogen Materials Advanced Research Consortium (HyMARC) in 2016 to address fundamental scientific barriers and support applied research.^235^ Materials-based storage can achieve higher hydrogen densities by physically or chemically binding hydrogen to other elements. For comparison, hydrogen density is approximately 111.8 kg m^–3^ in water at ambient conditions, 40 kg m^–3^ as a gas compressed to 700 bar, and 71 kg m^–3^ as a liquid at −253 °C. However, practical implementation requires storage materials with suitable thermodynamics, rapid hydrogen absorption and release kinetics, and acceptable gravimetric capacity. These technologies are generally classified as metal hydrides, adsorbent materials, and chemical hydrogen carriers.^227^ Carbon-based materials are promising hydrogen storage media because of their high surface area, which enables efficient hydrogen adsorption. Their storage performance can be further improved through approaches such as Kubas interactions, spillover effects, surface functionalization, alloying, and the incorporation of suitable additives.^236^

Hydrogen can be transported as compressed gas, liquid hydrogen, or in metal hydride form via road, pipelines, or maritime transport. Compressed hydrogen is suitable for short-distance, small-scale distribution, whereas pipelines are more efficient for transporting large volumes over long distances. Liquid hydrogen is primarily used for long-distance road and marine transportation because of its higher volumetric energy density, while metal hydride systems are generally limited to small-scale, short-distance applications due to their lower storage capacity and higher weight.^228^ Compressed hydrogen is typically transported in trucks or trailers using pressure vessels operating at 20–50 MPa. Horizontal storage tanks are generally mounted directly on trucks or trailers, whereas vertical hydrogen cylinders are secured in metal frames before transportation.^237^ Pipeline transportation is the most cost-effective option for moving large volumes of hydrogen over long distances.^228^ The widespread adoption of hydrogen as an alternative to fossil fuels will require the development of extensive pipeline infrastructure. However, the current dedicated hydrogen pipeline network remains limited and is mainly operated by industrial gas suppliers to deliver hydrogen to refineries and chemical plants.^237,238^ Hydrogen is typically transported over long distances by sea in liquid hydrogen, which offers economic advantages due to its high energy density and established transportation infrastructure. Emerging alternatives, such as liquid organic hydrogen carriers and metal-organic frameworks (MOFs), are also being explored for future overseas transport. However, liquefied hydrogen transportation remains challenging because hydrogen must be stored at 20 K (−253 °C) in specialized cryogenic vessels to ensure safety, in addition to the high energy required for liquefaction.^239,240^

## 7 Analysis of Challenges and Limitations in Green Hydrogen Production

Global green hydrogen production depends on the availability and quality of crucial raw materials alongside the advancement of technologies capable of plummeting environmental impacts. Although green hydrogen is broadly recognized as a clean energy carrier, hydrogen is produced by hydrocarbon reforming technologies. These conventional ways remain cost-effective and more commercially recognized than existing green hydrogen technologies, which are presently climbing toward economic competitiveness.

A central consideration in the evolution to green hydrogen is the availability and purity of input materials, mainly water, for electrolysis. Electrolysis needs high-quality water, and its sourcing demands careful evaluation of environmental and resource trade-offs. When evaluated against renewable surface water availability and broader water budgeting frameworks, water supply appears to be a significant constraint, especially in regions already facing water scarcity. In such circumstances, the water energy nexus becomes a critical factor as energy production progressively depends on water resources that are themselves under compression.

Past resource availability and existing hydrogen production strategies must also be scrutinized through the lens of long-term sustainability. This includes evaluating the security of raw material supply chains, the resilience and efficiency of production technologies, and their broader ecological implications. Ensuring that green hydrogen systems are not only sustainable but also environmentally responsible requires integrated planning that accounts for material inputs, regional resource limitations, and the growing environmental footprints of emerging technologies.

### 7.1 Water-Energy Nexus

The evolution toward a “green hydrogen economy,” in which hydrogen is formed and employed without accompanying greenhouse gas emissions, depends chiefly on electrolysis, powered by renewable electricity.^241^ With a decline in the cost of renewable energy and the electrolyzer technologies having improved in efficiency significantly, the focus has been transferred to the resource requirements (feedstocks), particularly the water availability, a major runner hydrogen driven green economy.^242^ Given the immediate transformation of the system and ecological shifts, concerns have been raised regarding the existence of water sources to withstand large-scale hydrogen production, specifically when estimating water usage across the whole production chain, containing its role as an important feedstock and as a cooling standard in thermoelectric methods such as steam methane reforming (SMR) as discussed above.^243^

However, the essential evidence of green hydrogen is the selected utilization of renewable energy pathways that do not necessitate cooling systems based on scarce fossil fuels.^244^ Hence, valuations of water demand should emphasize the water unswervingly used during the electrolysis. For example, when water use is limited to the electrochemical splitting stage alone, investigations specify that the overall water requirement for hydrogen production is relatively trivial compared to global freshwater accessibility.^245^ Subsequently, the direct water consumption related to green hydrogen production is likely to exert substantial pressure on global water resources available for the green hydrogen production industry.^246^

#### 7.1.1 HER Electrocatalysts Challenges

The hydrogen evolution reaction (HER) in alkaline environments is more difficult than that in acidic ones because it requires an extra water dissociation step, which slows down the reaction and increases overpotentials. In alkaline electrolytes, the HER performance depends not only on hydrogen adsorption and desorption but also on water adsorption, water splitting, and interactions between hydroxyl ions and the catalyst surface. As a result, many catalysts show lower activity under alkaline conditions. To overcome these issues, researchers have created bifunctional catalysts such as Pt-Ni­(OH)_2_ and Ni-Mn_3_O_4_, where one component aids water splitting, and the other supports hydrogen adsorption and release. Additionally, effective HER catalysts should be made from earth-abundant, low-cost metals or nonmetals and produced through simple, scalable methods to support large-scale hydrogen production.^156^

#### 7.1.2 Water Intake in Electrolysis

It has been anticipated that global demand for hydrogen among various sectors for the sake of a green economy is rising substantially, including chemical processes, fuel for transportation, heating systems of residential and commercial buildings, storage, and aviation.^247^ Approximately 2.3 Gt annually of global hydrogen requirement is anticipated in a renewable energy system in the future,^2^ supported by hydrogen produced by electrolysis powered exclusively by renewable energy sources. This remarkable evolution tends to minimize the energy sector’s carbon emissions by up to 10.2 Gt per year. These anticipations are substantially higher when compared with foreseeable emissions according to the Intergovernmental Panel on Climate Change (IPCC) RCP 8.5 scenario.^2^

However, the water requirements, an essential component to implement hydrogen-based energy systems, must be evaluated on the basis of available reservoirs. Various literature studies have shown concern over the economic and ecological feasibility of adequate water resources to support a global hydrogen economy.^248–250^ The significant issues must be addressed by estimating the total freshwater extraction and consumption in electrolysis for hydrogen production.^251^ Water extraction refers to the extraction, utilization, and return of water to the source, while consumption demonstrates the complete utilization of extracted water and conversion into a different form, i.e., hydrogen.^252^

Interestingly, hydrogen production is very low in cost-effectiveness, as the water-to-hydrogen ratio is approximately 9:1, as shown in the equation:^253^

Hydrogen production from water splitting has the following stoichiometric dynamics:

1 $$2H_{2}O\to 2H_{2}+O_{2}$$

Molecular weight of molecules and compounds in grams/mol

Oxygen: 15.999 g/mol

Hydrogen: 1.008 g/mol

$$water\;H_{2}O:\;\left(\left(\left(1.008\right)\times 2\right)+\left(15.999\right)\right)=18.015\;g/mol\;...\;\left(input\right)$$

$$hydrogen\;gas\;H_{2}:\;\left(1.008\right)\times 2=2.016\;g/mol\;...\;\left(output\right)$$

Therefore, the theoretical ratio is about:

Production Ratio = Input/Output

= (18.015)/(2.016) ≈ 8.937 ≈ 8.94 litres of water required to produce 1 kg of hydrogen

Based on the stoichiometric production of hydrogen gas from a molecule of water, the mass fraction of approximately 11.19% hydrogen^248^ is produced from one water molecule, as shown in the equation:

$$m\mathrm{ass}\;\mathrm{fraction}\;\mathrm{of}\;\mathrm{hydrogen}\;\left(\%\right):\;\frac{H_{2}}{H_{2}O}\times 100\approx \frac{2.016}{18.015}\times 100=\approx 11.19\%$$

However, the mass fraction of hydrogen from water molecules does not clearly present the concept of hydrogen production efficiency. The production efficiency, on the other hand, is based on the ratio of the amount of energy contained in the hydrogen molecule (heating value) and the electrical energy supplied to split the water molecules (electrolyzer system).^254^

$$\eta_{electrolyzer}=\frac{energy\;contained\;in\;hydrogen}{electrical\;energy\;supplied}\times 100\%$$

The heating value of hydrogen depends on the quality and latent heat of water during the time of industrial processing. The high heating value (HHV) assumes the condensed water vapors, capturing total chemical energy, whereas the low heating value (LHV) eliminates the latent heat of water vaporization.^255^ The literature reports that the energy efficiency of industrial electrolyzers ranges between 60–80% HHV with energy consumption between 3.35-5.6 kWh/Nm^3^.^256^

By the year 2030, the hydrogen production is anticipated to reach the mark of 5.7 million tonnes,^257^ which, if sourced from water splitting, would require a whopping 50.94 million cubic meters of fresh water. Additionally, the regenerated water in the process, such as hydrogen fuel cells and combustion, is not returned to the source, thus it is considered as consumed.^258^ The only division in which hydrogen employment does not completely regenerate water is chemical synthesis, anticipated to necessitate 540 Mt of hydrogen yearly, equivalent to a maximum freshwater requirement of around 4.8 billion m^3^ per year, or roughly 0.3 ppm of worldwide freshwater disposal.^2^

#### 7.1.3 Water Demand and Sector Comparison

When equated with other forecasts of water demand related to hydrogen production, the freshwater requirements projected here are low. This conclusion principally echoes the fundamental postulation that future hydrogen production will be powered completely by renewable energy sources, such as wind and solar, which need negligible to no freshwater for electricity production.^259^ In contrast, when fossil fuels are utilized for primary energy production and power generation, water supplies are considered considerable. On the other hand, it was found that about 251 billion cubic meters of freshwater were employed for fossil fuel-based energy production and power generation, such as natural gas, coal, and crude oil, out of which approximately 31 billion cubic meters of water were used in cooling, refining, and mineral processing industries.^260^ By evaluation, while the 20.5 billion m^3^ of water needed for hydrogen production through electrical splitting of water (electrolysis) would be completely consumed, the following quantities remain at 33% less than the existing fossil fuel-based water consumption (Figure 9). Furthermore, a large-scale shift to electrolytic hydrogen production could gradually relocate energy systems based on fossil fuels.^261^ With the shift of the energy sector toward emerging renewable technologies, a projected 10 billion cubic meters of freshwater reservoirs could be avoided, which must be spent on fossil fuel-based interventions.

**Figure 9:**
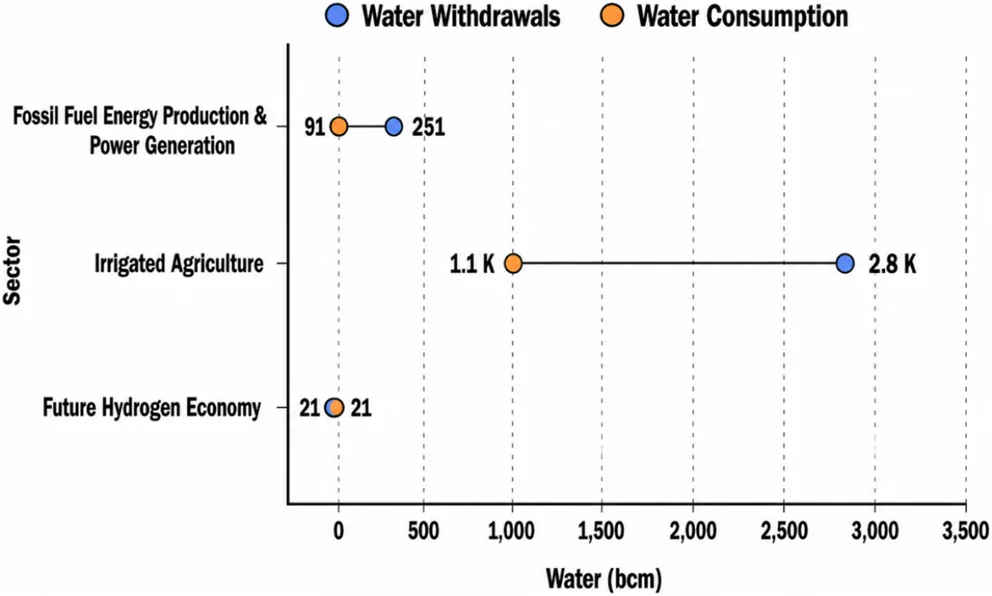
Global freshwater extraction and consumption across different sectors 10 years ago.

A recent anticipated shift in global water withdrawals between 2021 and 2030 under a decarbonization pathway^262^ consistent with a net-zero transition, exhibited freshwater budgeting across the energy sector (Figure 10). Water withdrawals related to primary fossil fuel production reduce significantly to 37.5%, dropping from roughly 320 to 200 billion cubic meters, whereas fossil fuel–based power generation declines by around 40%, from roughly 250 to 150 billion cubic meters. These drops imitate the taper-down of natural gas, coal, and oil withdrawal and the equivalent narrowing of thermoelectrical power generation, both of which are exceedingly water-intensive due to coal mining, processing, and cooling needs. Conversely, water withdrawals for hydrogen production surge distinctly (+300%), increasing from around 10 to 40 billion cubic meters as electrolytic hydrogen production grows quickly. Bioenergy also displays substantial growth of about 75%, growing from around 40 to 70 billion cubic meters, expectedly driven by increased agricultural activities and biomass production^263^ increments are anticipated in nuclear power to about 14.3% and renewables of about 20% in the future, suggesting incremental bulk extension, while numerous renewable technologies, such as solar and wind photovoltaics, have reasonably low operational water needs.

**Figure 10:**
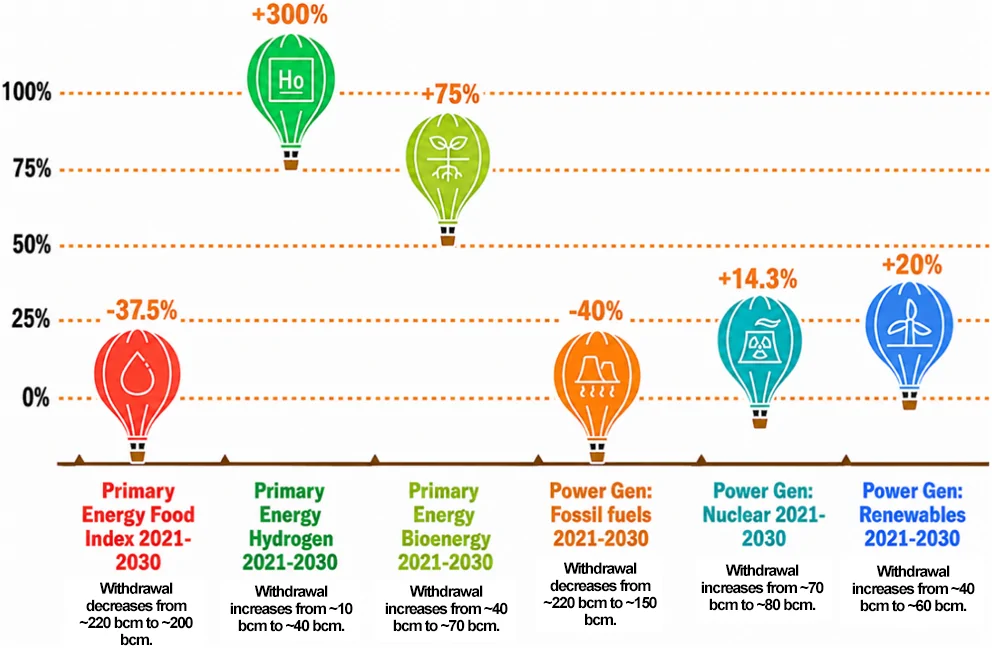
Anticipated global freshwater withdrawals (2021-2030) in the energy and power generation sector.

However, compared to water consumption in the agricultural sector, the water expenditure in hydrogen production by electrolysis is minimal. Globally, the agriculture sector receives about 70% of global freshwater for food security purposes, summing up to a whopping 2700 billion cubic meters yearly. Not to mention, over irrigation in the aridland agriculture, where scorching climatic conditions encourage farmers to over irrigate the crops to prevent dry topsoil and eventual desiccation of the rooting zone.^264^ Additionally, the management of landscaping with turf grass causes the irrigation of grasses with no economic value, and biomass ends up in compost, eventually adding to the carbon footprint.^265^ Resultantly, 1100 billion cubic meters of freshwater are consumed annually, which is 50 times more than anticipated for green hydrogen production, as shown in Figure 9.

However, despite the minimal demand for freshwater resources for the electrolytic splitting of water into green hydrogen, it is still a significant quantity as compared to the agricultural sector aiming for food security. Nevertheless, the green economy saves the planet, and agriculture saves lives (Alam, unpublished). Though growing interest and concerns toward the green economy among planet managers and researchers, freshwater water budgeting has become the utmost need among all sectors for survival and sustainability. Ultimately, there is complete or partial shifting of the green economy and green hydrogen production from electrolysis of the desalinated ocean waters.

#### 7.1.4 Limitations of Water Sources for Green Hydrogen Production

To ensure the decarbonization of hard-to-abate, carbon-intensive industrial subdivisions including transport, chemicals, steel, and fertilizers, the production of green hydrogen via renewable-based water electrolysis is a crucial phenomenon. The large-scale implementation of water-based green hydrogen production requires ample inputs of electrical energy and a purified water supply.^266^ In this case, the waste and seawater costs monetary and economic strain for the transition to green energy. As discussed earlier, the theoretical water supply of about 9 litres of pure water produces one kg of green hydrogen, though based on life cycle assessment, the total water input in running the complete process is substantially higher due to inclusion of purification, cooling, balancing the plant operations, and cleaning.^267^ With the extent of advanced electrolyzer capacities and net-zero clean energy production approaches, a sustainable map of water management for green hydrogen production is crucial and must be examined multidimensionally.

The availability of freshwater denotes a major concern because, apparently, the parts of the world with remarkable and well-managed renewable energy potential lack the availability of quality, clean freshwater. The coding behind the water channels opens up multiple reasons for water stress, including overpopulation, industrial wastewater dumping, global climate shifts, and microplastic pollution.^268^ Almost 4 billion individuals incur water shortages and drought for most of the year, whereas rapid urbanization, industrial interventions, and intensive production units impose substantial strain on freshwater resources.^269^ Such societal and environmental programs initiate a competitive scenario between large-scale green hydrogen production units and crucial sectors such as domestic water use, agricultural water use, industrial water intake, and ecological water requirements, raising a perilous scientific question about sustainable water management for native and invasive sectors running on water.

Such incidents seem significantly important in the hyperarid regions of the world, such as the Middle East and North African countries (MENA), some parts of Southeast Asia, the United States of America, and the deserts of Australia. These regions experience intensive solar and wind-driven energy production units that rely on limited freshwater resources.^270^ The International Renewable Energy Agency (IRENA) has published several reports, emphasizing the consideration of freshwater feedstock for the establishment of green hydrogen production projects, especially in water-challenged regions as mentioned above.^271^

However, the availability of freshwater reservoirs and the purity of water as an input feedstock for green hydrogen production are not similar. For instance, the commercial proton exchange membrane electrolyzers (PEMs) demand the input of graded purified water.^272^ This is due to the high risk of catalyst and membrane damage because of traces of dissolved salts, heavy metals, mineral ions such as magnesium, calcium, chloride, and suspended debris, thus reducing the life of the production unit with significantly reduced efficiency.^273^ Because of such risks, the water from freshwater reservoirs is passed through a filtration process such as ion exchange, distillation, reverse osmosis, or complete deionization. Eventually, such deionization systems add high cost for the equipment installations, and the rate of water purification is also slow, thus reducing the hydrogen production efficiency per unit time.^274^

Ocean and seawater present a desirable alternative due to their ample abundance of water, reducing stress on freshwater reservoirs, and lower anthropogenic pollution rates to support the green hydrogen production units for the arid regions sharing a coastline, such as Australia, and MENA regions. The proximity of coastlines to arid regions substantially reduces the transportation costs and alleviates the net-zero production phenomenon.^275^ However, untreated seawater is not suitable for the water electrolyzers used for green hydrogen production. Ocean water bears high electrical conductivity due to dissolved salts, which most likely poses a risk to the maintenance of the electrolyzers. The chloride anions can accelerate collateral reactions, leading to chlorine gas production that contributes to corrosion. Cations such as calcium and magnesium tend to form scales on the membranes and electrodes of the electrolyzer, requiring regular replacements and cleaning.^276^ However, there are research projects involved in the electrolysis of raw seawater, though the outcomes are represented by insignificant data.^277^

Conventionally, arid regions rely on the reverse osmosis (RO) process to desalinate seawater for domestic, agricultural, and industrial use.^278^ Similarly, the electrolysis of seawater is conducted after the reverse osmosis of seawater for green hydrogen production, although it seems to be an intensive process. RO is an advanced technology commonly used in Middle Eastern regions that nearly purifies water by eliminating dissolved salts, suspended solids, microorganisms, and environmental contaminants.^279^ In terms of energy inputs and expenditure, the TEA and LCA analyses have demonstrated that the RO process adds significantly less energy as compared to the water electrolysis systems. The research has shown that approximately tens of kilowatt-hours of electrical energy is required to produce a kilogram of hydrogen, while the desalination of about 9 litres of water as feedstock to produce 1 kg of green hydrogen adds up to 3–4 kilowatt-hours of energy.^280^

However, a substantial limitation of the desalination process is the secondary production of concentrated salt solution (brine) that requires additional management. Unmanaged discharge of brine can lead to the degradation of the environment, with increasing local salinity levels and the addition of salinity pollutants to the land and marine environmental components. The global strategies formed by organizations emphasize the controlled release of brine to designated locations, with further valorization strategies under investigation to attain net-zero clean energy processes for green hydrogen production.^281^

#### 7.1.5 GCC Vulnerability to the Green Economy

Middle Eastern and neighboring countries have arid climatic conditions that significantly hinder life on land. The scarce water resources and high atmospheric temperatures make the saline sandy soils even drier and hamper agricultural activities. With the population increasing to about 50 million in recent years, the expected population by 2050 is 14 million higher. According to the Orient Planet Report (OPR), the water demand for the GCC countries will hit 33,733 million cubic meters by 2050, given that the storage opportunities of about 25,855 million cubic meters are insufficient, needing to expand by 77 percent for optimal usage for the next 30 years^282^ (Figure 11).

**Figure 11:**
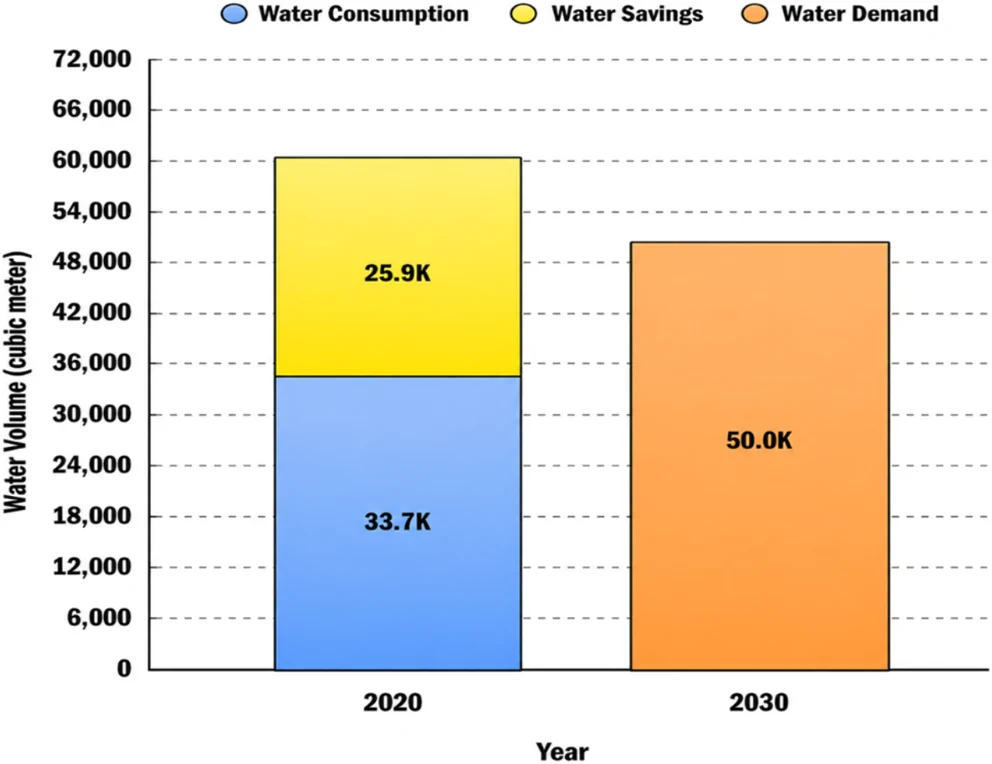
Projected water consumption, demand, and storage volumes of GCC countries (2030–2050).

In GCC regions, with an annual rainfall below 100 mm, the total renewable surface water is about 4.14 billion cubic meters annually. Approximately 71.3% of the total water share is produced from renewable resources, and desalination contributes only 23.7%. Agriculture accounts for about 70–80%, the largest share, for food security, signifying the higher investments in water for agriculture as compared to the green economy.^283^ As briefly discussed above, the tweak in freshwater ratios can hamper the energy and food supply chain sectors. For example, energy is needed throughout the food value chain, making the interface between food for energy and energy for food particularly significant. Additionally, food bills are strongly affected by energy expenses, exaggerated by price increases, and fuel market disruptions.^284^ Also, the water-food affiliation is well recognized as water availability strongly influences agricultural interventions. The scarcity of freshwater resources to meet future demands, arid regions like GCC and neighboring countries rely on desalination of water and reclaimed water, such as treated sewage effluent, for urban agriculture, which impose significant risks.^285^ However, the appropriate use of recycled water is economically beneficial to levitate the green economy and manage climate change altogether.

The GCC countries are dynamically aligning themselves to become chief performers in the global hydrogen economy, exploiting their profuse renewable energy potential and the prevailing energy infrastructure. The global hydrogen demand is expected to range around 530 million tonnes (Mt) annually by 2050, accounting for crudely 7% of global primary energy utilization.^286^ Inside this wider development, the GCC and North African regions are anticipated to generate about 4 Mt of hydrogen per year by 2030 for trade-level hydrogen production. The United Arab Emirates has pronounced some of the most tangible production goals. The UAE intends to generate about 1.4 Mt of hydrogen annually by the year 2031, with determinations to measure up to approximately 15 Mt per year over the next 25 years. For the future, UAE targets launching two hydrogen hubs by the year 2031 and developing five more by the year 2050.^287^ On the other hand, the Kingdom of Saudi Arabia KSA has correspondingly innovated immense hydrogen projects, such as the NEOM green hydrogen and ammonia project, anticipated to be a main trade leader and exporter of green ammonia in the coming years.^288^ The NEOM establishment embodies one of the biggest global green hydrogen projects and indicates the KSA’s intention to lead as a global energy driver.

The GCC’s relative lead lies chiefly in its access to low-cost renewable electricity, mainly solar power. High solar irradiance levels permit the region to possibly produce green hydrogen at internationally competitive prices, given that renewable energy costs continue to reduce. Preserving the cost benefit will be vital, as the global hydrogen market infiltration will rely profoundly on cost competitiveness compared to conventional technologies offered by other global players.

#### 7.1.6 Desalination of Salt Water: An Alternative

Freshwater resources are a critical limitation in producing green hydrogen, mainly in arid regions, because agriculture claims most of it. On the basis of the limited efficiency of green hydrogen production per 9 liters of water, it further constrains the large-scale production of green hydrogen for the levitation of the green economy in the water-scarce regions. Recent numbers of water withdrawals are anticipated to rise from 2.23 to 12.09 billion cubic meters, and consumption is expected to rise from 1.72 to 8.32 billion cubic meters for the green hydrogen production in the next 25 years.^289^ As discussed earlier, the diversion of freshwater resources to green hydrogen production may initiate a catastrophic competition between the municipal and agricultural sectors, thus necessitating the finding of substitutes for freshwater for the electrolytic production of green hydrogen.

Arid regions such as GCC countries situated near seacoasts represent the major producers of desalinated water. The total desalinated water capacity of the GCC countries alone is about 4.14 billion cubic meters annually, indicating that these countries account for 57% of the global desalinated water producers.^290^ Therefore, the countries have the potential to employ a scalable water source to produce green hydrogen. The extreme ecological conditions of limited freshwater resources, proximity to the coastline, and reliance on desalinated water highlight a logical integration point with green hydrogen value chains. In the municipal sector, the high-purity water produced by reverse osmosis and thermal desalination is regularly employed, which can further be adopted to source pure water for alkaline electrolyzers and proton exchange membrane technologies of green hydrogen production.^291^

Extensive life cycle assessment (LCA) research investigations on different pathways of using reclaimed water for green hydrogen production declared desalinated water as a realistically scalable option for quality and marketable green hydrogen production. However, the desalination of water for sourcing green hydrogen production comes with notorious limitations and ecological impacts (Figure 12). Unfortunately, desalination imposes significant ecological impacts, given the high-energy-driven process, expensive membrane installations, washing, maintenance, replacements, and brine disposal.^292^ Major apprehension is exhibited with regard to carbon footprints, raising sustainability concerns, as one solution leads to another problem. Nevertheless, a major research gap and an endeavor for future research lies in whether the existing strategy, coupled with renewable electricity and continuous green hydrogen production, could reduce the environmental burden substantially. Brine discharge exhibits a significant shortcoming of the desalination process. When disposed of into the ocean, it imposes a significant increase in the electrical conductivity of the ocean water, threatening marine life and reducing the water quality for further desalination, making it challenging and intensive.^293^ Retreatment of the brine by virtue of mitigation strategies demands energy and byproducts that can lethally deteriorate the environment and climate.

**Figure 12:**
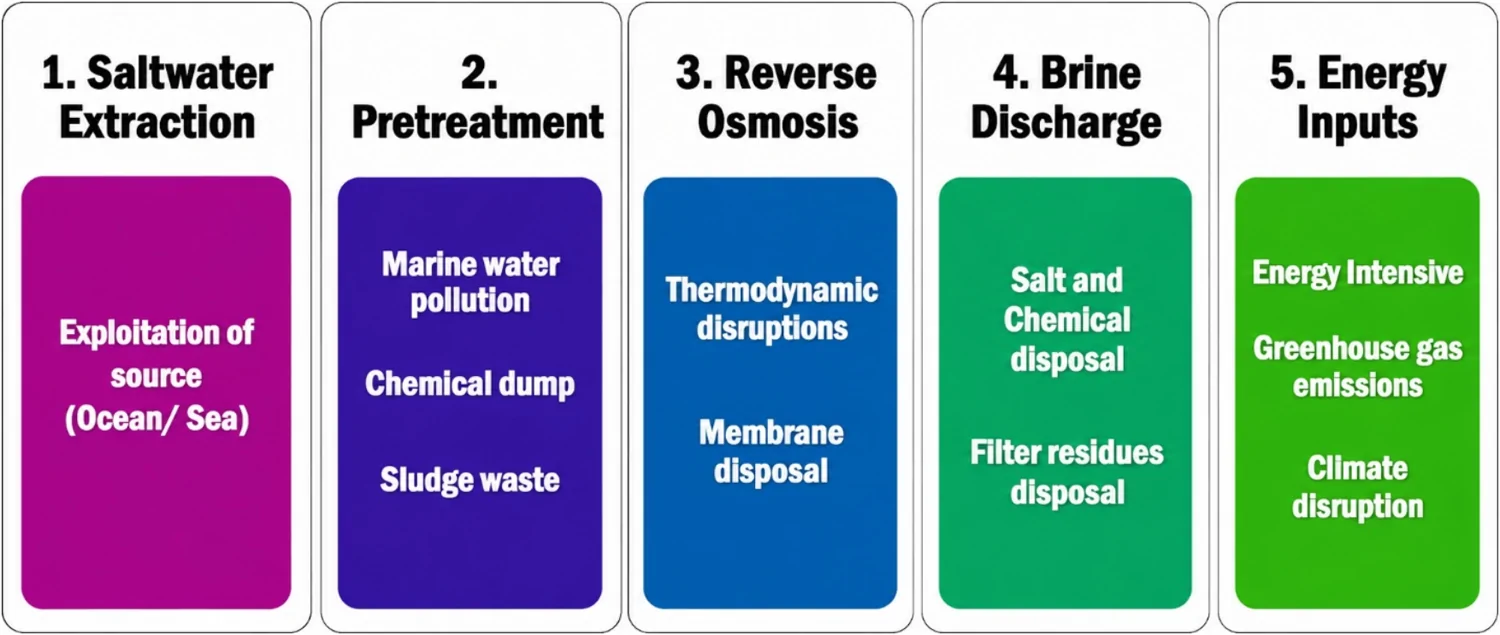
Ecological impact of the desalination process.

Besides the drawbacks and significant energy inputs and ecological impacts, desalination offers a viable approach to sourcing high-purity water for green hydrogen production, particularly in the GCC countries. The exploitation of freshwater sources is relatively the least impactful when compared to the desalination potential of arid lands, when coupled with renewable energy systems. Maybe it also involves fewer energy trade-offs and procures more benefits from green hydrogen production and global trades.

## 8 Sourcing of Biomass and Renewable Energy for Hydrogen Production

Biomass waste is a promising option for hydrogen production through thermochemical processes such as gasification and pyrolysis, offering a simultaneous waste management solution instead of landfilling.^200^ However, its commercialization remains limited because of the complexity and heterogeneity of biomass, which complicates the development of well-controlled systems capable of handling variations in feedstock characteristics. This often necessitates physical and chemical pre-treatment of the biomass. Post-treatment is also required for syngas purification. Moreover, biomass transportation and large areas are needed for waste storage, which introduces additional cost.^4^ Integrating concentrated solar energy as a heat supply for thermochemical hydrogen production is an attractive approach. However, the dependence on the weather and the intermittent nature of solar energy complicate its large-scale implementation,^294,295^ suggesting the incorporation of backup or energy storage systems.^63^ Hydrogen production from biomass via fermentation faces several limitations related to the sensitivity of process parameters, such as pH, temperature, and nutrient availability. Deviations from these conditions can reduce the hydrogen yields. Additionally, feedstock variation and the difficulty in maintaining stable microbial consortia complicate scalability.^73^ In photocatalytic water splitting techniques, advanced semiconductor-based photocatalysts with a wide range of light absorption and new approaches for preventing photogenerated charge recombination, as well as new techniques for gas separation, must be investigated to achieve scalability.^173^ Current photocatalytic systems achieve less than 1% solar-to-hydrogen efficiency, which is less than that of solar-driven electrolysis.^16^

The large-scale implementation of water electrolysis plants powered by renewable energy sources such as solar and wind is limited by the fluctuations of these resources; thus, a hybrid hydrogen production system enables continuous hydrogen generation by utilizing two energy sources, thereby reducing the impact of intermittency.^296^ For the AWE system, the major challenges are the low current density and its limited ability to adapt to the variable power input.^108^ Further research is needed to develop durable and cost-effective anode and cathode materials as well as more conductive electrolytes to reduce cell resistance.^112^ In the PEM system, reducing the cost while maintaining the efficiency is challenging.^108^ Moreover, research should focus on developing a robust and stable electrocatalyst at higher current densities in PEM water electrolysis.^119^ In the SOE system, the main challenge restricting commercialization is material degradation, such as intergranular cracking, densification, and cation migration, resulting from the high operating temperatures. Therefore, further studies should address improving the performance and stability of SOE cell materials by refining the fabrication methods and enhancing casting techniques. Additionally, operating at lower temperatures by selecting materials with lower activation energies can help reduce thermal-stress-induced degradation.^116^

Solar thermochemical water splitting for hydrogen production is one of the technologies that have strong potential for large-scale deployment. However, achieving scalability requires that several limitations be overcome. The development and optimization of redox materials that are thermally stable and can be easily reduced. Also, it is required to reduce the energy consumed by the mechanical vacuum pumps for oxygen removal from the reduction chamber, which can be achieved by implementing cascading-pressure reduction chambers. The current efficiency of the thermochemical cycle ranges between 5 and 10%, which can be enhanced by advancing new redox materials, a more efficient oxygen pumping system, and the recovery of the cycle’s excess heat for electricity generation.^107,297^

In addition to the aforementioned challenges associated with green hydrogen production technologies, the infrastructure needed for hydrogen storage and transport is expensive and requires further development. For instance, hydrogen pipelines must be specifically engineered to ensure safe and efficient transport. This often necessitates the use of specialized materials, which increase the infrastructure costs. Moreover, compressing hydrogen for storage and transport requires high energy input, further raising the overall cost of hydrogen transportation.^298^

## 9 Conclusions

Green hydrogen provides notable benefits over traditional fossil fuels by supporting environmental sustainability and decarbonization. Consequently, many countries have been exploring green hydrogen production methods. Incorporating renewable energy sources into processes such as photocatalytic and thermochemical water splitting, biomass gasification, and water electrolysis is essential, as it offers emission-free energy and lowers costs. Nonetheless, the intermittent nature of these renewable resources hampers their widespread use, emphasizing the importance of strategies such as thermal storage systems to mitigate this challenge. AWE and PEM are the most advanced electrolysis technologies. Currently, AWE cells are the most cost-effective among electrolysis methods, but further research is needed to boost their efficiency. PEM systems require more affordable catalysts, while SOE advantages include the use of seawater and potential coupling with renewable energy sources for steam production. AEM is less studied but addresses high PEM costs and the low current density associated with the AWE. For the commercialization of water electrolysis, it is essential to incorporate non-noble-metal-based electrocatalysts to address the scarcity and high expense of noble metals. Photocatalytic water splitting holds promise for large-scale applications due to its simplicity; however, effective hydrogen and oxygen separation, reduced charge recombination, and improved light harvesting are essential, as the current efficiency remains lower compared to that of solar-driven electrolysis. Waste-to-hydrogen methods using technologies like gasification and fermentation, which utilize biomass, wastewater, and industrial byproducts, offer promising avenues for waste valorization and sustainable hydrogen production. However, these technologies are limited by the availability of biomass, its storage, and the variability in its composition, which affect the yield.

Despite extensive research on green hydrogen technologies, scalability is still hindered by technical and economic challenges. Water quality and availability constitute significant constraints for hydrogen production via electrolysis, particularly given the intake of 8.94 L of water to produce 1 kg of hydrogen, with a mass fraction of 11.19% hydrogen from the electrical splitting of water in electrolyzer systems, underlining the substantial water input required. The higher energy efficiency of hydrogen production (60–80%) in electrolyzer systems can be achieved under ideal conditions of the HHV of hydrogen in water molecules. These compressions also raise concerns regarding potential ecological degradation, especially in water-scarce regions. However, green hydrogen-based energy systems offer prominent environmental benefits, including mitigation of greenhouse gas emissions and reduction of climatic catastrophes. Therefore, future endeavors encourage the use of recycled or desalinated water as alternative feedstocks have been increasingly discussed and promoted as a feasible strategy to support sustainable green hydrogen production. Future research should focus more on integrating artificial intelligence (AI) with computational methods, such as density functional theory (DFT), to streamline electrocatalyst design and screening, thereby speeding up the development and commercialization of green hydrogen technologies. Through research and development investments coupled with international collaboration, green hydrogen has the potential to emerge as a reliable and scalable energy source.

## Data Availability

Data sharing is not applicable to this article as no data were created or analysed.
